# Do You Read How I Read? Systematic Individual Differences in Semantic Reliance amongst Normal Readers

**DOI:** 10.3389/fpsyg.2016.01757

**Published:** 2016-11-22

**Authors:** Anna M. Woollams, Matthew A. Lambon Ralph, Gaston Madrid, Karalyn E. Patterson

**Affiliations:** ^1^Neuroscience and Aphasia Research Unit, University of ManchesterManchester, UK; ^2^MRC Cognition and Brain Sciences UnitCambridge, UK; ^3^Department of Clinical Neurosciences, University of CambridgeCambridge, UK

**Keywords:** reading, semantics, phonology, acquired dyslexia, connectionist modeling, individual differences

## Abstract

The extent to which meaning is involved in reading aloud has proven an area of longstanding debate, and current computational models differ on this dimension. The connectionist triangle model proposes that normal individuals rely on semantic information for correct reading of words with atypical spelling-sound relationships, but to varying degrees. This proposed individual difference would account for the varying stage of decline at which patients with semantic dementia first show the reading impairment known as surface dyslexia. Recent neuroimaging data has provided validation of this view, showing that individual differences in degree of semantic reliance during exception word reading predict the amount of activation in left anterior temporal regions associated with semantic processing. This study aimed to establish the cognitive correlates of individual differences in semantic reliance during exception word reading. Experiment 1 used a subgrouping approach with 32 participants and found larger imageability and semantic priming effects specifically for exception word reading amongst high relative to low semantic reliance readers. High semantic reliance readers also tended to read nonwords more slowly than low semantic reliance readers. A second experiment used a regression approach with 129 readers and confirmed the relationship of degree of semantic reliance both to imageability effects in exception word reading and speed of nonword reading. Further, while the performance of the higher semantic readers revealed no significant association with semantic processing tasks, there was a negative relationship with rhyme processing tasks. We therefore speculate that differences in phonological abilities may be responsible for varying degrees of semantic reliance in reading aloud. This proposal accords with the results of functional imaging showing that higher semantic reliance during exception word reading corresponds to lower activation in left pre-central gyrus, an area associated with direct spelling sound mapping and phonological processing. Our results therefore establish the nature of systematic individual differences in degree of semantic involvement amongst normal readers, and suggest directions for future neuroimaging and computational modeling research to uncover their origins.

## Introduction

Although there is general agreement that the process of normal reading aloud involves use of a combination of sub-word and whole-word procedures to map between spelling and sound, the nature of each of these procedures remains controversial. According to the localist dual-route computational model (Coltheart et al., 2001), nonwords (novel letter strings) are read aloud via a system of grapheme-phoneme correspondence rules within a nonlexical route, and exception words (with atypical spelling-sound correspondences) require processing via a direct lexical route, while an additional unimplemented lexical-semantic pathway can provide access to word meaning. In contrast, a distributed connectionist model based on learnt probabilistic associations (Plaut et al., 1996) has demonstrated that both nonwords and exception words could be read aloud correctly via a single direct pathway between spelling and sound. When a semantic pathway was included in this model during learning, then the lower-frequency exception words came to rely upon activation of meaning for their correct pronunciation. Yet the role of semantic processing in exception word reading has remained controversial due to a failure to observe this selectivity in large scale studies of normal reading (Balota et al., 2004) and variability in exception word reading impairments amongst patients with semantic deficits (Blazely et al., 2005). Recent neuroimaging data has supported the proposal that there are considerable individual differences in the degree of semantic reliance during exception word reading (Hoffman et al., 2015). In this paper, we harness the semantic dimensions of imageability and semantic priming to understand the nature of these individual differences in degree of semantic reliance amongst normal readers.

Initial investigations of individual differences in normal reading aloud were framed entirely within an unimplemented dual-route framework. Baron and Strawson (1976) were the first to demonstrate the impact of spelling-sound typicality [in terms of the extent to which words obeyed the pronunciation rules of Veneszky (1970)] upon word reading speed in normal participants (Experiment 1). They also considered the extent to which readers may rely on a subword (which they called orthographic) procedure or a whole-word (which they called lexical) procedure, leading them to contrast the performance of two groups of “Phoenician” and “Chinese” readers respectively (Experiment 2). Phoenician readers were those who were good at determining whether the pronunciation of a nonword was identical to a known word. Chinese readers were those who were good spellers and able to recognize correct spellings in a forced choice test. As predicted, the Phonecian readers showed a larger difference between regular and exception words (processed via subword and whole-word mechanisms respectively) than Chinese readers (for whom all items were thought to be translated to phonology via the whole-word mechanism). Baron and Strawson considered these results to provide good evidence for the functional independence of the subword and whole-word procedures.

The distinction between Phoenician and Chinese readers was later pursued by Brown et al. (1994), following the dual-route logic that nonwords were read correctly by the nonlexical route, exception words were read correctly via the direct lexical route, and regular words were read correctly via either route. In this study, nonword homophone decision, spelling to dictation and forced choice spelling tasks were used to classify readers as Phoenician or Chinese. The predictions for the Phoenicians were that they should show faster nonword reading RTs and smaller effects of the number of syllables (the latter was assumed to reflect greater automaticity of nonlexical functioning) plus a smaller advantage for words (i.e., a reduced lexicality effect) in a mixed list of regular words and nonwords. These predictions were supported overall, but not when subgroups matched on word reading times were considered. Chinese readers, in contrast, were predicted to have faster exception word naming latencies and smaller frequency effects for these items (the latter was assumed to reflect greater automaticity of lexical functioning) plus a smaller advantage for regular words over exception words; but the results revealed the opposite pattern.

Given the somewhat inconsistent findings of initial explorations into normal individual differences in reading aloud, attention turned to neuropsychology as a means to reveal the functional architecture of the reading system (Patterson and Hodges, 1992; Patterson and Marcel, 1992). At the same time, a connectionist model of reading aloud demonstrated that all words could be processed via a single direct pathway between spelling and sound (Seidenberg and McClelland, 1989). Initially, this approach had difficulty in accounting for strong dissociations in nonword and exception word reading ability seen in some patients (Patterson et al., 1996). Indeed, the need to account for the selective deficits in exception word reading observed in surface dyslexia was a key motivation for extension of the connectionist account to implement an approximation of the semantic pathway necessary for activation of word meaning (Plaut et al., 1996).

The inclusion of a version of the semantic pathway in the connectionist triangle model allowed a good simulation of surface dyslexic reading due to the fact that a division of labor emerged in the model over the course of training which maximized the efficiency of the overall functioning of the model. As learning progressed, the direct nonsemantic pathway came to specialize in mappings that were high in frequency and/or typicality, because the model could then fall back on the whole-word activation available from the semantic pathway to ensure correct pronunciations of items low in frequency or typicality and especially both. Due to this division of labor in the model, when it was lesioned by reducing the semantic contribution to phonology in order to approximate impaired semantic processing, the model produced the selective deficit in exception word reading that defines surface dyslexia, with the errors corresponding to the regularizations made by the patients (e.g., *pint* read to rhyme with *mint*).

While the division of labor simulations in the triangle model demonstrated its ability to account for surface dyslexia, it also seemed to entail a very strong prediction: all patients who have semantic deficits should also have surface dyslexia. This prediction did seem to fit many patients within the literature, particularly those with semantic dementia (SD), a selective progressive deficit of semantic knowledge associated with atrophy of the anterior temporal lobes (Patterson and Hodges, 1992; Graham et al., 2000). Yet this pattern was not universally observed: a few SD patients were reported in the literature with a dissociation between their impaired semantic knowledge and intact exception word reading (e.g., Cipolotti and Warrington, 1995; Blazely et al., 2005).

Traditional neuropsychology emphasizes the importance of single cases of dissociation over larger scale association observed in case-series studies. Hence the existence of even one patient with impaired semantic knowledge but preserved exception word reading was held to demonstrate that the close relationship between the two abilities seen in many other patients was merely an accident of anatomical contiguity: areas involved in exception word reading might be adjacent to those involved in semantic memory, such that most lesions would affect both. Thus, even a few dissociations between semantic and exception word reading abilities were argued to disprove the validity of the connectionist account of surface dyslexia (Blazely et al., 2005; Coltheart et al., 2010).

One reason that traditional neuropsychology can place such a heavy weight upon evidence from single cases is because it is assumed that any differences between individuals in terms of their cognitive abilities/processing styles before they suffered brain damage are likely to exert a negligible impact on post-lesion performance (Shallice, 1988). There is, however, evidence that people's pre-morbid experience and knowledge does appreciably influence their performance after brain damage (e.g., Jefferies et al., 2011), and this would seem to be particularly likely with a later acquired skill like reading. This led Plaut (1997) to propose that dissociations between semantic knowledge and exception word reading might result from variations in the degree of division of labor within the reading system. His simulations demonstrated that versions of the connectionist triangle model trained with strong semantic support developed a marked division of labor, such that when the semantic contribution was reduced to approximate SD, severe surface dyslexia was the result. In contrast, those versions of the model trained with weak semantic support developed a milder division of labor, meaning that when the semantic contribution was reduced, exception word reading was spared, producing the pattern seen in the key single dissociation cases.

This idea was pursued by Woollams et al. (2007) in the context of a large case-series study of reading aloud in SD. Their computational simulations involved training multiple versions of the connectionist triangle model with varying semantic support and then lesioning them by reducing and disrupting semantic activation to differing degrees to emulate the progressive decline in conceptual knowledge that characterizes SD. The patient data showed an overwhelming association between degree of conceptual deterioration (on semantic tests not involving reading) and degree of impaired exception word reading, mimicking the simulation data. Nevertheless, there was considerable variation between different patients in terms of the extent of the reading deficit seen at any given level of semantic deficit. Indeed, three of the SD cases (out of 51), initially had intact low-frequency exception word reading. Yet, when followed longitudinally, all three cases progressed into surface dyslexia. Within the context of a larger case series, these temporary dissociations appear to represent extremes within a normal distribution. Woollams et al. (2007) therefore proposed that the amount of semantic damage needed to produce surface dyslexia in a given individual at least in part reflected their pre-morbid degree of reliance on semantic information for exception word reading. Further simulation work has explored the extent to which pre-morbid individual differences in reading exposure and direct pathway resources can impact upon the correspondence between performance on semantic tasks and exception word reading (Dilkina et al., 2008).

The division of labor hypothesis makes the clear prediction that semantic effects in normal reading aloud should be most apparent for words with exceptional spelling sound mappings. One semantic dimension that has been often studied in the context of reading aloud is that of imageability, which is usually measured by ratings of the extent to which a word's referent evokes a mental image (Paivio et al., 1968). Connectionist models have incorporated the dimension of imageability into their semantic representations in terms of either variation in the number of semantic features (Plaut and Shallice, 1993) or the degree of intercorrelation between semantic features (Harm and Seidenberg, 2004). In either case, semantic activation of phonology during reading aloud is stronger for high than low imageability words. This then confers a performance benefit to high over low imageability words. In line with the division of labor hypothesis, Harm and Seidenberg (2004) demonstrated that imageability produced its strongest effects on the reading of low frequency exception words within their instantiation of the connectionist triangle model. This accurately simulates previous research showing significant effects of imageability only upon exception word reading in normal readers (Strain et al., 1995; Shibahara et al., 2003; Woollams, 2005).

A similar result is obtained when the impact of semantic priming upon reading aloud is examined. In this paradigm, the extent to which reading of a given word is facilitated by prior presentation of a semantically related word related to an unrelated word is assessed. Cortese et al. (1997) demonstrated stronger semantic priming effects for exception than regular words, as expected according to the division of labor account. Semantic priming effects on lexical decision have been simulated within connectionist models through assuming that related primes share semantic features and often co-occur with the target (Plaut and Booth, 2000), although the impact of semantic priming on reading aloud has yet to considered.

Although reported in multiple studies, the interaction between regularity and imageability predicted by the division of labor account has failed to replicate in large scale megastudies of reading aloud (Balota et al., 2004). The hypothesis that there are individual differences in the degree of semantic reliance offers an explanation of why the effects of semantic variables for exception words may prove unreliable, as they would only be appreciable for a subset of participants.

As to which participants might be expected to show the strongest effects of meaning level variables on exception word reading, Hoffman et al. (2015) developed a behavioral measure to tap the extent to which individuals relied upon the semantic vs. direct pathway. Low imageability words provide weaker semantic activation of phonology than high imageability words by virtue of fewer features with lower intercorrelations (Plaut and Shallice, 1993; Harm and Seidenberg, 2004), hence performance on these items should best reveal the operation of the direct pathway. Readers who rely little on semantics for exception word reading should be able to process words with both typical and atypical spelling-to-sound relationships primarily via their direct pathway, and should therefore show small consistency effects for low imageability items. In contrast, readers who rely strongly on semantics will have to wait for slower additional whole-word activation from meaning to support pronunciation of atypical words, and hence these readers should show large consistency effects for low imageability items. In a crucial link between individual differences in normal reading and the neuropsychological data, Hoffman et al. (2015) found that this behavioral index predicts the amount of activation during reading in an area of the left lateral anterior temporal lobe that responds more strongly to exception than regular words, consistent with the idea that it measures the degree of an individual's semantic reliance during exception word reading.

The aims of this study were to establish the cognitive correlates of the semantic reliance (SR) index introduced by Hoffman et al. (2015). Firstly, we tested the prediction that performance on this measure should correspond to variation in the semantic effects of imageability and semantic priming, as these have both been shown to exert their influence specifically on exception word reading. Secondly, we investigated whether the SR index corresponds to differences in the representation of subword mappings along the direct pathway by considering performance on nonword reading, as this is a task that allows assessment of orthography to phonology conversion without semantic influence. Thirdly, we considered how the SR index corresponds to performance in written and spoken semantic and phonological tasks, in order to determine if variation in semantic reliance in reading is linked to individual differences in language processing more generally. We explored these issues across two experiments, the first adopting a focused subgrouping approach to word and nonword reading and the second adopting a larger scale regression approach to semantic and phonological processing.

## Experiment 1

Experiment 1 considered whether the reading aloud performance seen for low SR and high SR readers varies in principled ways according to the predictions of the connectionist triangle model. Firstly, we would expect the high SR readers to show a larger effect of imageability than low SR readers for words with atypical spelling-sound correspondences, but with no such differences apparent for typical words. Secondly, we sought convergent evidence of differences in semantic reliance using a semantic priming task, with the expectation that high SR readers should show larger effects of semantic priming for atypical words than low SR readers, but again with no difference for typical words. Thirdly, we attempted to assess associated differences in the structure of the direct route suggested by the division of labor hypothesis by considering nonword reading performance. To the extent that the high SR readers have a direct pathway specialized to consistent spelling-sound mappings, then they may be more likely than low SR readers to produce the most common pronunciations for nonwords with inconsistent bodies. Moreover, as high SR readers would have relatively little competition from alternative spelling-sound mappings along the direct pathway, the nonword reading times of such individuals might also be characterized by a smaller consistency effect than seen for low SR readers.

### Method

#### Participants

Thirty two members of the MRC Cognition and Brain Sciences Unit volunteer panel were paid for their participation in this study. All were native speakers of British English, aged between 18 and 40 years. The research was approved by the Cambridgeshire Local Research Ethics Committee.

#### Stimuli

The properties of the four stimulus sets used in the present study are summarized in Tables 1–**4**.

**Table 1:** **Average values (and standard deviations) for the imageability word set on a range of psycholinguistic variables**.

	**Consistent**		**Inconsistent**	
	**High imageability**	**Low imageability**	**High imageability**	**Low imageability**
Imageability	552.55 (47.23)	381.60 (60.19)	552.58 (53.68)	380.6 (46.44)
Age of acquisition	5.95 (2.03)	8.30 (2.73)	5.96 (2.08)	7.71 (3.00)
SUBTLEX frequency	73.65 (150.15)	138.52 (435.04)	125.56 (295.16)	126.11 (240.57)
KF frequency	80.63 (143.47)	87.20 (155.85)	82.55 (153.35)	85.4 (116.07)
Letter length	4.35 (0.74)	4.60 (0.59)	4.43 (0.84)	4.53 (0.78)
Phonemic length	3.30 (0.52)	3.45 (0.64)	3.30 (0.72)	3.28 (0.68)
Body neighbors	11.90 (6.53)	12.08 (6.56)	10.38 (6.52)	13.08 (8.56)
No. friends	11.90 (6.53)	12.08 (6.56)	3.28 (3.08)	4.5 (4.62)
Summed friend frequency	677.80 (1331.97)	659.28 (837.87)	325.70 (465.15)	535.95 (818.56)
No. enemies	0.00 (0.00)	0.00 (0.00)	7.10 (4.47)	8.58 (5.05)
Summed enemy frequency	0.00 (0.00)	0.00 (0.00)	1356.40 (3347.08)	1059.28 (1288.23)
Type consistency ratio	1.00 (0.00)	1.00 (0.00)	0.32 (0.15)	0.33 (0.15)
Token consistency ratio	1.00 (0.00)	1.00 (0.00)	0.39 (0.30)	0.37 (0.31)

##### Imageability

The first set of items consisted of a factorial manipulation of spelling-sound consistency and imageability, with 40 monosyllabic items per cell, and are listed in Appendix A (Supplementary Material). Items consisted of quartets across the conditions that were matched in terms of their initial phoneme. We chose to manipulate spelling-sound consistency rather than grapheme-phoneme regularity as the former has shown to be more influential upon word reading performance in skilled adults (Cortese and Simpson, 2000). All of the consistent words were regular according to the nonlexical route of the DRC model (Coltheart et al., 2001) and perfectly consistent in that they had orthographic bodies that were always pronounced in the same way (Jared, 1997, 2002), meaning that they all had a type and token consistency ratio (number of friends/number of friends-number of enemies and summed frequency of friends/summed frequency of friends-summed frequency of enemies respectively) of 1 (Ziegler et al., 1997). The inconsistent words all had orthographic bodies that were pronounced with a different rime in at least one other word, with 29 of the low imageability and 28 of the high imageability items also being irregular according to the nonlexical route of the DRC model.

The properties of the stimuli are displayed in Table 1 and were analyzed using a series of 2 (consistency) by 2 (imageability) ANOVAs. Imageability ratings (Paivio et al., 1968) showed only the expected main effect of imageability [*F*_(1, 156)_ = 431.84, *p* < 0.0005], with no reliable effects of consistency or any interaction [*F*s_(1, 156)_ < 0.004, *p*s > 0.951]. Unsurprisingly, Age of Acquisition ratings (Kuperman et al., 2012) showed a main effect of imageability [*F*_(1, 151)_ = 26.42, *p* < 0.0005], with no reliable effects of consistency or any interaction [*F*s_(1, 151)_ < 0.576, *p*s > 0.449]. The significant difference across imageability on Age of Acquisition within our stimuli reflects the correlation between these two measures (McFalls et al., 1996). We are of the view that age of acquisition effects emerge from the operation of the semantic pathway, due to differences in the semantic representation (Brysbaert et al., 2000) or the links between phonology and semantics (Lambon Ralph and Ehsan, 2006). Hence, as per imageability, age of acquisition provides an index of the strength of semantic activation of phonology during reading, which is the dimension we aimed to capture with our index of semantic reliance.

No significant differences across conditions were apparent for: SUBTLEX word form (Brysbaert and New, 2009) frequency [*F*s_(1, 154)_ < 0.047, *p*s > 0.494]; (Kucera and Francis, 1967) frequency [*F*s_(1, 156)_ < 0.043, *p*s > 0.835]; number of letters [*F*s_(1, 156)_ < 2.21, *p*s > 0.139]; number of phonemes [*F*s_(1, 156)_ < 0.739, *p*s > 0.391]; and orthographic body neighborhood size [*F*s_(1, 156)_ < 1.64, *ps* > 0.202]. Number of friends and summed KF frequency of friends showed the expected main effects of consistency, although only marginally significantly so for the latter [*F*_(1, 156)_ = 90.05, *p* < 0.0005; *F*_(1, 156)_ = 2.77, *p* = 0.098], with no reliable effects of imageability or any interaction [*F*s_(1, 156)_ < 0.673, *p*s > 0.413]. Number of enemies and summed KF frequency of enemies also showed the expected main effects of consistency [*F*_(1, 156)_ = 215.90, *p* < 0.0005; *F*_(1, 156)_ = 18.15, *p* < 0.0005], with no reliable effects of imageability or any interaction [*F*s_(1, 156)_ < 0.191, *p*s > 0.169]. Lastly, both type and token consistency ratio showed significant main effects of consistency [*F*_(1, 156)_ = 1643.14, *p* < 0.0005; *F*_(1, 156)_ = 329.29, *p* < 0.0005], but no main effect of imageability or interaction [*F*s_(1, 156)_ < 0.072, *p*s > 0.789].

##### Priming

The second set of items consisted of a factorial manipulation of spelling-sound consistency and semantic priming, with 20 targets and primes per cell, and are listed in Appendix B (Supplementary Material). No target or prime items overlapped with the imageability set, and all were of medium imageability according to the Cortese and Fugett (2004) norms. Targets were monosyllabic and were paired to related primes using the Maki et al. (2006) norms. Within consistency, two sets were constructed, such that for one participant, Set A would appear with related primes, while Set B would appear with unrelated primes (formed by repairing primes and targets in the set), while for the next participant, this assignment of sets to relatedness conditions would be reversed. All but three of the consistent targets were regular according to the nonlexical route of the DRC model (Coltheart et al., 2001) and all were perfectly consistent with a type and token consistency ratio of 1. The inconsistent words all had orthographic bodies that were pronounced with a different rime in at least one other word, with 27 items also being irregular according to the nonlexical route of the DRC model.

With respect to the properties of the targets, displayed in Table 2, a series of 2 (consistency) by 2 (set) ANOVAs showed no significant differences across conditions for: imageability ratings [*F*s_(1, 76)_ < 1.39, *p*s > 0.243]; Age of Acquisition ratings [*F*s_(1, 76)_ < 2.27, *p*s > 0.136]; SUBTLEX word form frequency [*F*s_(1, 76)_ < 0.826, *p*s > 0.366]; CELEX (Baayen et al., 1993) written frequency [*F*s_(1, 76)_ < 1.79, *p*s > 0.185]; number of letters [*F*s_(1, 76)_ < 1.87, *p*s > 0.175]; number of phonemes [*F*s_(1, 76)_ < 0.028, *p*s > 0.867]; and orthographic body neighborhood size [*F*s_(1, 76)_ < 0.618, *p*s > 0.434]. Number of friends and summed CELEX frequency of friends showed the expected main effects of consistency [*F*_(1, 76)_ = 70.18, *p* < 0.0005; *F*_(1, 76)_ = 7.24, *p* = 0.009], with no reliable effects of set or any interaction [*F*s_(1, 76)_ < 0.790, *p*s > 0.377]. Number of enemies and summed CELEX frequency of enemies also showed the expected main effects of consistency [*F*_(1, 76)_ = 55.76, *p* < 0.0005; *F*_(1, 76)_ = 24.32, *p* < 0.0005], with no reliable effects of set or any interaction [*F*s_(1, 76)_ < 0.223, *p*s > 0.638]. Lastly, both type and token consistency ratio showed significant main effects of consistency [*F*_(1, 76)_ = 694.95, *p* < 0.0005; *F*_(1, 76)_ = 106.16, *p* < 0.0005], but no main effect of set or interaction [*F*s_(1, 76)_ < 2.31, *p*s > 0.133].

**Table 2:** **Average values (and standard deviations) for the semantic priming word targets on a range of psycholinguistic variables**.

	**Consistent**		**Inconsistent**	
	**Set A**	**Set B**	**Set A**	**Set B**
Imageability	5.14 (1.28)	5.15 (1.31)	5.54 (1.03)	5.38 (1.23)
Age of acquisition	6.18 (1.71)	5.79 (1.30)	5/70 (2.04)	6.62 (2.46)
SUBTLEX frequency	42.46 (72.38)	60.21 (66.20)	59.19 (97.40)	45.87 (65.40)
CELEX frequency	41.52 (57.01)	54.11 (86.97)	73.51 (90.77)	41.32 (57.88)
Letter length	4.35 (0.81)	4.45 (0.51)	4.40 (0.60)	4.70 (0.66)
Phonemic length	3.25 (0.55)	3.25 (0.64)	3.25 (0.72)	3.30 (0.73)
Body neighbors	11.20 (5.79)	10.35 (5.00)	9.70 (5.85)	9.75 (7.06)
No. friends	11.20 (5.79)	10.35 (5.00)	3.40 (2.28)	2.60 (2.28)
Summed friend frequency	685.19 (618.43)	666.29 (686.14)	353.11 (480.69)	302.85 (501.24)
No. enemies	0.00 (0.00)	0.00 (0.00)	6.30 (4.62)	7.15 (6.60)
Summed enemy frequency	0.00 (0.00)	0.00 (0.00)	480.36 (513.24)	441.25 (659.52)
Type consistency ratio	1.00 (0.00)	1.00 (0.00)	0.39 (0.14)	0.32 (0.17)
Token consistency ratio	1.00 (0.00)	1.00 (0.00)	0.47 (0.32)	0.42 (0.36)

The properties of the primes are displayed in Table 3. A series of 2 (consistency) by 2 (set) ANOVAs showed a marginally significant set effect for SUBTLEX word form frequency [*F*s_(1, 76)_ = 2.94, *p* = 0.090], but no differences across consistency or any interaction [*F*s_(1, 76)_ < 0.109, *p*s > 0.742], and there were not any significant differences for CELEX written frequency [*F*s_(1, 76)_ < 1.60, *p*s > 0.210]. No significant differences were seen across conditions for: number of letters [*F*s_(1, 76)_ < 0.676, *p*s > 0.414]; number of syllables [*F*s_(1, 76)_ < 0.831, *p*s > 0.365]; forward association strength from prime to target [*F*s_(1, 76)_ < 1.90, *p*s > 0.173]; backward association strength from target to prime [*F*s_(1, 76)_ < 2.41, *p*s > 0.125], and semantic distance between prime and target computed from WordNet [*F*s_(1, 76)_ < 1.27, *p*s > 0.264] and using LSA [*F*s_(1, 76)_ < 4.03, *p*s > 0.709].

**Table 3:** **Average values (and standard deviations) for the semantic priming word primes on a range of psycholinguistic variables**.

	**Consistent**		**Inconsistent**	
	**Set A**	**Set B**	**Set A**	**Set B**
SUBTLEX frequency	26.57 (25.69)	57.14 (127.81)	24.29 (26.70)	69.46 (145.87)
CELEX frequency	30.13 (29.43)	42.79 (87.73)	26.71 (34.35)	50.85 (84.86)
Letter length	4.85 (1.04)	5.15 (1.18)	5.15 (1.50)	5.30 (1.13)
Syllable length	1.25 (0.44)	1.40 (0.50)	1.45 (0.51)	1.40 (0.50)
Forward association	0.17 (0.20)	0.23 (0.18)	0.23 (0.24)	0.16 (0.16)
Backward association	0.08 (0.20)	0.09 (0.13)	0.17 (0.24)	0.12 (0.18)
WordNet semantic distance	1.75 (2.68)	1.57 (2.05)	2.71 (2.49)	1.82 (2.37)
LSA semantic distance	0.31 (0.18)	0.28 (0.18)	0.31 (0.21)	0.35 (0.20)

##### Nonwords

The third set of items consisted of 160 nonwords based on the consistency by imageability word set described above and are listed in Appendix C (Supplementary Material). In order to ensure that initial phoneme matching was preserved across consistency, these nonwords were formed by swapping onsets within each list of 40 items. Statistics concerning the consistency of the pronunciation of the nonwords, as presented in Table 4, were computed assuming the “regular” nonword pronunciation as produced by the nonlexical route of the DRC model (Coltheart et al., 2001). A series of *t*-tests confirmed that the consistent and inconsistent body nonwords did not differ on letter length, phonemic length, body neighborhood and summed KF frequency of friends [*t*_(1, 158)_ < 0.849, *p* > 0.397] and did differ on number of friends, number of enemies, summed KF frequency of enemies, and type and token feedforward consistency ratios [*t*_(1, 158)_ > 3.63, *p* < 0.0005].

**Table 4:** **Average values (and standard deviations) for the consistent and inconsistent body nonwords on a range of psycholinguistic variables**.

	**Consistent**	**Inconsistent**
Letter length	4.46 (0.71)	4.48 (0.86)
Phonemic length	3.38 (0.64)	3.29 (0.66)
Body neighbors	12.00 (6.47)	11.70 (7.67)
No. friends	12.00 (6.47)	6.73 (5.11)
Summed friend frequency	668.41 (1105.73)	665.19 (1155.89)
No. enemies	0.00 (0.00)	4.98 (4.02)
Summed enemy frequency	0.00 (0.00)	973.46 (2399.64)
Type consistency ratio	1.00 (0.00)	0.54 (0.23)
Token consistency ratio	1.00 (0.00)	0.44 (0.33)

#### Procedure

Participants completed the three tasks (reading aloud of the imageability words, the semantic priming words, and the nonwords) across two sessions, separated by at least 2 weeks. In one session they completed the imageability by consistency and semantic priming by consistency word lists, always in this order to prevent biasing a semantic processing strategy by presentation of related primes in the first task. The two versions of the priming task were alternated according to the order of enlistment. In order to prevent priming due to the overlap of orthographic bodies, the nonword list was completed in a separate session. The order of sessions was counterbalanced across participants.

For the imageability by consistency word reading and matched nonword reading tasks, participants were instructed to read the letter string aloud as soon as possible while avoiding errors. Each trial involved presentation of a white fixation cross for 500 ms, followed by the letter string in lower case until response, up to a maximum of 2000 ms, using DMDX software (Forster and Forster, 2003). Latencies were derived using a microphone mounted on a headset via a voicekey algorithm. Any mistriggers and accuracy of response were monitored by the experimenter. Responses to each item were also recorded into .wav files for later offline response coding. The next trial then appeared after 250 ms. Participants completed 12 representative practice trials before completing the main block of 160 items, which were randomized anew for each participant. The procedure for the eight practice and 80 critical items for the semantic priming word reading task was identical, with the exception that a 500 ms uppercase prime intervened between the fixation cross and the target word.

### Results

#### Imageability

**Figure 1:**
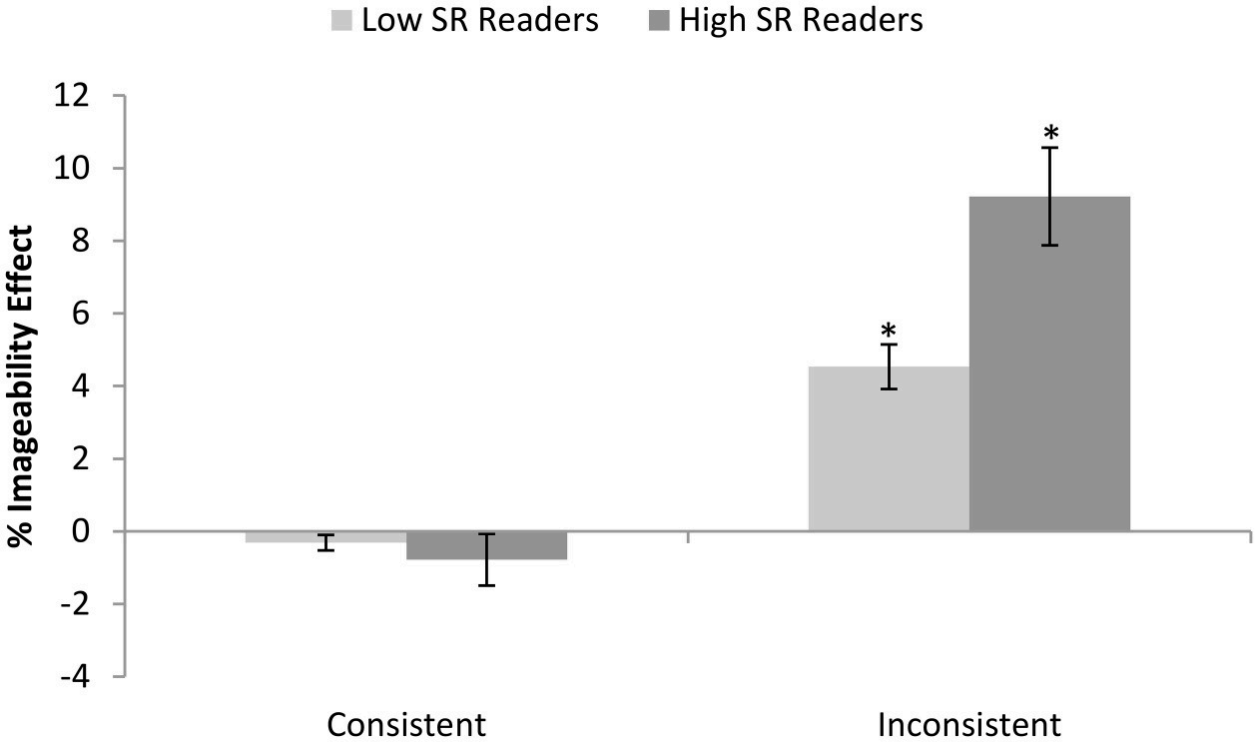
**Percentage imageability effect seen in error rates for each reader group (high vs. low semantic reliance) according to consistency**. Error bars represent standard error. Asterisks indicate significant imageability effects of *p* < 0.0005.

We first needed to quantify each person's degree of semantic reliance for exception word reading on the SR index. To achieve this, we computed inverse efficiency measures (Röder et al., 2007; Roberts et al., 2010) for each condition by dividing the correct reaction time with the proportion correct, then computed the consistency effect (inconsistent-consistent) for the low imageability items. The 16 participants with the smallest consistency effect were deemed low SR readers and the 16 with the largest consistency effect high SR readers. Cell means and standard deviations of correct reaction times and error rates for each group are provided in Table 5. We conducted a 2 (between: reader group) by 2 (within: consistency) by 2 (within: imageability) ANOVA for each dependent variable. The results revealed a main effect of group, indicating faster and more accurate responses for the low than high SR readers [*F*_(1, 30)_ = 7.06, *p* = 0.013; *F*_(1, 30)_ = 13.40, *p* = 0.001]. There were main effects of consistency [*F*_(1, 30)_ = 25.81, *p* < 0.0005; *F*_(1, 30)_ = 111.40, *p* < 0.0005] and a consistency by group interaction [*F*_(1, 30)_ = 21.93, *p* < 0.0005; *F*_(1, 30)_ = 18.14, *p* < 0.0005], reflecting the larger consistency effects for the high than low SR readers. There were main effects of imageability [*F*_(1, 30)_ = 7.61, *p* = 0.010; *F*_(1, 30)_ = 440.67, *p* < 0.0005], and an imageability by group interaction in errors [*F*_(1, 30)_ = 53.17, *p* = 0.017], due to a larger imageability effect for the high than low SR readers. There was a significant interaction between consistency and imageability in errors [*F*_(1, 30)_ = 320.36, *p* < 0.0005] and critically, the three way interaction between group, consistency and imageability was also significant in errors [*F*_(1, 30)_ = 35.60, *p* = 0.001]. This indicated that, as predicted, high SR readers showed a significantly bigger imageability effect than the low SR readers for the inconsistent words [*t*_(30)_ = 3.17, *p* = 0.003], but not for the consistent words [*t*_(30)_ = 0.63, *p* = 0.533]. As can be seen in Figure 1, both groups showed significant imageability effects for the inconsistent but not consistent words.

**Table 5:** **Average values (and standard deviations) of RT and error rates for the low and high SR readers according to consistency and imageability**.

		**Low SR readers**		**High SR readers**	
		**RT**	**Errors**	**RT**	**Errors**
Consistent	High image	546 (88)	0 (0)	613 (101)	0.94 (1.55)
	Low image	546 (82)	0.31 (0.85)	632 (119)	1.72 (3.26)
Inconsistent	High image	542 (85)	0.63 (1.12)	644 (100)	3.13 (3.82)
	Low image	552 (89)	5.16 (2.32)	663 (119)	12.34 (6.49)

#### Priming

**Figure 2:**
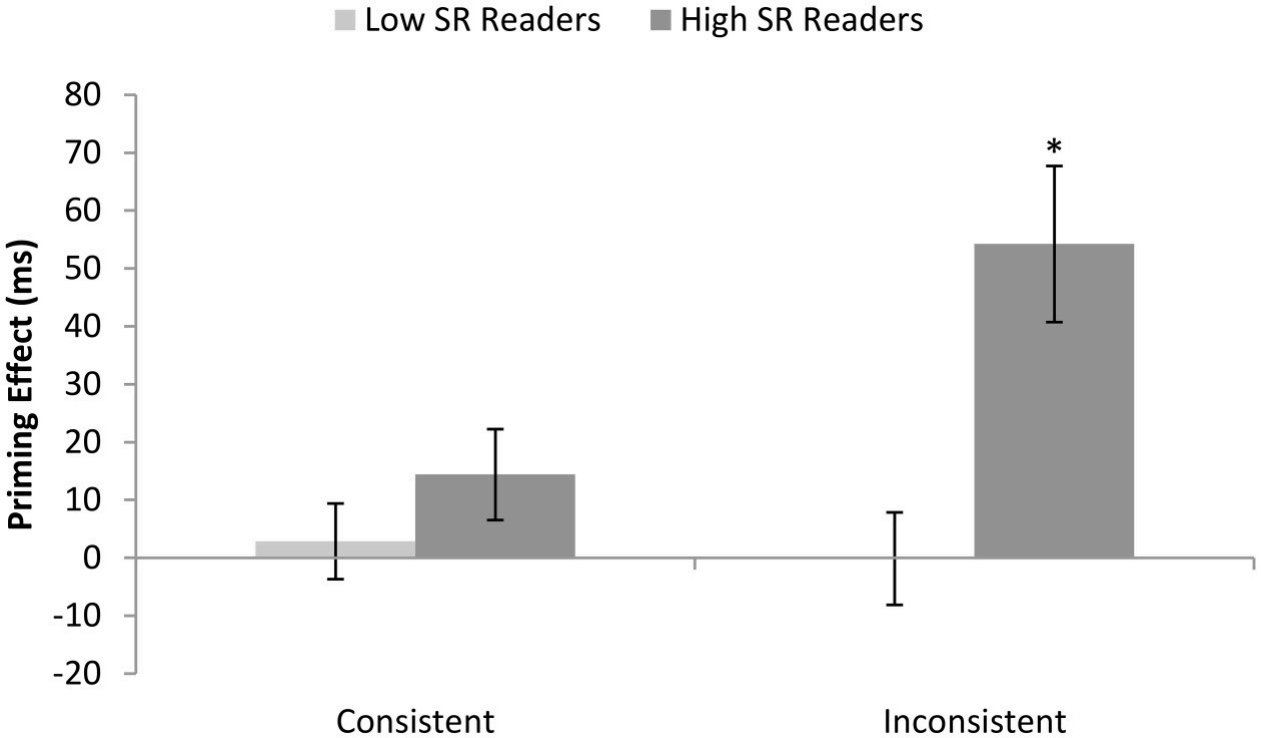
**Average priming effect seen in reaction times for each reader group (high vs. low semantic reliance) according to consistency**. Error bars represent standard error. Asterisks indicate significant priming effects of *p* < 0.005.

We considered the impact of the same subgrouping of low and high SR readers (based on the size of the consistency effect in inverse efficiency for low imageability words) upon the size of the semantic priming effects observed for consistent and inconsistent words. Cell means and standard deviations of correct reaction times and error rates for each group are provided in Table 6. A 2 (between: reader group) by 2 (within: consistency) by 2 (within: priming) ANOVA was conducted on each variable. There were main effects of consistency in both reaction times and error rates [*F*_(1, 30)_ = 56.18, *p* < 0.0005; *F*_(1, 30)_ = 19.23, *p* < 0.0005] and a consistency by group interaction in reaction times [*F*_(1, 30)_ = 13.85, *p* = 0.001], reflecting the larger consistency effects for the high than low SR readers. Reaction times also showed a main effect of priming [*F*_(1, 30)_ = 19.47, *p* < 0.0005], and a priming by group interaction [*F*_(1, 30)_ = 16.61, *p* < 0.0005], due to a larger priming effect for the high than low SR readers. There was a marginally significant interaction between consistency and priming in reaction times [*F*_(1, 30)_ = 3.09, *p* = 0.089], but most importantly, the three way interaction between group, consistency and priming was also significant [*F*_(1, 30)_ = 4.18, *p* = 0.050]. This indicated that high SR readers showed a significantly bigger priming effect than the low SR readers for the inconsistent words [*t*_(30)_ = 3.47, *p* = 0.002], but this did not hold for the consistent words [*t*_(30)_ = 1.13, *p* = 0.268]. As can be seen in Figure 2, the low SR readers did not show any reliable priming effect, whereas the high SR readers showed significant priming effects for inconsistent but not consistent words.

**Table 6:** **Average values (and standard deviations) of RT and error rates for the low and high SR readers according to consistency and priming**.

		**Low SR readers**		**High SR readers**	
		**RT**	**Errors**	**RT**	**Errors**
Consistent	Related	544 (95)	0.31 (1.25)	568 (100)	0.63 (1.71)
	Unrelated	547 (87)	0.63 (1.71)	582 (100)	0.94 (2.72)
Inconsistent	Related	557 (94)	1.25 (2.24)	581 (92)	3.44 (5.69)
	Unrelated	557 (76)	3.13 (4.03)	635 (121)	4.06 (3.28)

#### Nonwords

As the proportion of words containing a particular body-rime mapping has been found to be the strongest determinant of nonword reading performance (Andrews and Scarratt, 1998), we ranked the possible pronunciations for any given nonword according to the number of word types containing that body-rime correspondence, and the pronunciation containing the most common correspondence was considered to be the “consistent” one. A full list os pronunciations is provided in Appendix D (Supplementary Material). Of course, the consistent nonwords contained bodies that correspond to only one rime. Cell means and standard deviations are provided in Table 7. A 2 (between: reader group) by 2 (within: consistency) ANOVA on the percentage of nonword responses corresponding to the body pronunciation of one or more real words revealed (a) a main effect of group [*F*_(1, 30)_ = 5.21, *p* = 0.030]: fewer such “extant” pronunciations were given by high than low SR readers; and (b) a main effect of consistency [*F*_(1, 30)_ = 27.82, *p* < 0.0005], as more of these responses were seen for consistent than inconsistent nonwords, with no significant interaction between the two factors. A 2 (between: reader group) by 2 (within: pronunciation type) ANOVA on the type of body pronunciation given for inconsistent nonwords revealed a main effect of pronunciation type [*F*_(1, 30)_ = 1479.24, *p* < 0.0005], but contrary to expectation, no significant interaction with reader group. A 2 (between: reader group) by 2 (within: consistency) ANOVA on the RTs for consistent nonword pronunciations revealed a marginally significant main effect of group [*F*_(1, 30)_ = 3.00, *p* = 0.093], due to the slower RTs for high than low SR readers, and a significant main effect of consistency [*F*_(1, 30)_ = 15.37, *p* < 0.0005]; but again, the interaction between group and consistency was not significant.

**Table 7:** **Percentage extant body-rime pronunciations and reaction times for the most common body pronunciation for the low and high SR readers according to consistency (standard deviations given in parentheses)**.

		**Low SR readers**	**High SR readers**
Consistent	% body pronunciation	95.78 (6.16)	88.05 (12.81)
	Consistent RT	613 (123)	711 (182)
Inconsistent	% body pronunciation	77.31 (2.52)	72.69 (7.68)
	Consistent RT	642 (123)	728 (166)
	Proportion 1	0.48 (0.043)	0.506 (0.052)
	Proportion 2	0.507 (0.046)	0.482 (0.055)
	Proportion 3	0.013 (0.021)	0.011 (0.011)
	Proportion 4	0.001 (0.003)	0.001 (0.003)

*For inconsistent words, the proportion of each type of body pronunciation is also provided*.

*Proportion 1 is that for the most common body-rime mapping, Proportion 4 is the least common body-rime mapping*.

### Discussion

The goal of Experiment 1 was establish the cognitive correlates of variations in the degree of semantic reliance during exception word reading in terms of semantic effects for words and nonword pronunciations. We computed an index of semantic reliance based on the size of the consistency effect seen for low imageability items during reading aloud. This was based on the rationale that low SR readers with a small consistency effect read all words primarily via their direct pathway, while high SR readers with a large consistency effect require more semantic activation for correct reading of atypical words. Hence we would expect high SR readers to show a larger effect of imageability than low SR readers for inconsistent words, but not consistent words, and this was indeed the case in errors. It should be noted that this difference is not an artifact of the use of values for low imageability words in defining the SR index, as the high SR readers could have also have performed more poorly than the low SR readers on the high imageability inconsistent words, which would have produced only a two-way interaction between consistency and group. Moreover, we also found that high SR readers showed a larger effect of semantic priming in reaction times than low SR readers for inconsistent words, but not consistent words. These findings confirm that our measure taps degree of semantic involvement specifically for items with atypical spelling-sound mappings.

The division of labor account also suggests that differential semantic reliance during reading aloud for atypical words should have consequences for the structure of the direct pathway, because it allows this to specialize to common and consistent spelling-sound mappings, maximizing the performance of the system as a whole. We sought evidence for this proposal by comparing the nonword reading performance of the two types of readers. The results indicated that the high SR readers were less likely than low SR readers to produce nonword pronunciations corresponding to an extant body-rime mapping for both inconsistent and also consistent words, suggesting some difficulty with using higher order systematicities between spelling and sound. Contrary to expectations, we did not find any evidence for specialization of the direct pathway of high SR readers to consistent mappings: both groups produced similar proportions of consistent pronunciations for inconsistent nonwords, and the magnitudes of their RT consistency effects for nonwords were comparable, although the high SR readers tended to be generally slower.

Overall then, this experiment confirms that the SR index based on the size of the consistency effect for low imageability words seems to provide a valid marker for higher semantic reliance specifically for reading words with atypical spelling-sound mappings, as high SR readers showed significantly larger imageability and semantic priming effects than low SR readers specifically for these items. Yet high SR readers did not show any evidence of a direct pathway that had specialized to consistent body-rime mappings. In fact, they tended to show poorer performance on nonword reading in general, irrespective of consistency. Given the established link between nonword reading and phonological processing ability (Pennington et al., 1990), this result suggests we might see some individual differences in phonological processing tasks that correspond to the SR index. In addition, it may be that the larger semantic effects observed for high SR readers in exception word reading are associated with superior semantic processing ability. We explored this issue using written and spoken semantic and phonological processing tasks in a larger sample in Experiment 2.

## Experiment 2

The goal of Experiment 2 was to identify cognitive correlates of the differences in degree of semantic reliance during reading aloud in a large group of normal readers in nonreading language tasks. Reading acquisition builds upon the previously developed primary language systems of semantics and phonology (Patterson and Lambon Ralph, 1999; Harm and Seidenberg, 2004). Experiment 2 therefore used both written and spoken tests of semantic and phonological processing. It is possible that a higher degree of semantic reliance during reading aloud is associated with relatively strong semantic processing and/or relatively weak phonological processing. Individuals with poor reading comprehension have deficits in written synonym judgment and also in spoken category fluency (Nation and Snowling, 1998), hence we chose to use these tasks to tap semantic processing. Developmental dyslexics, on the other hand, show deficits in written rhyme judgment (Hoeft et al., 2006) and spoken rhyme fluency (Fraser et al., 2010), hence we chose to use these tasks to tap phonological processing. Our expectations were that high SR readers would show better performance on the semantic tasks of synonym judgment and category fluency, and/or poorer performance on the phonological tasks of rhyme judgment and rhyme fluency, none of which require any reading aloud.

### Method

#### Participants

One hundred and twenty nine University of Manchester undergraduate psychology students participated in this study for course credit. All were native speakers of British English, aged between 18 and 40 years. The research was approved by the University of Manchester Research Ethics Committee.

#### Stimuli

For the Imageability and Nonword tasks, the stimuli used were identical to those from Experiment 1.

##### Judgment tasks

Semantic processing was tapped by computerized administration of the Synonym Judgment test from the PALPA (Test 50, Kay et al., 1992). This test consists of 30 pairs of items, half high imageability (e.g., shovel-spade) and half low imageability (e.g., menace-threat), with items in the two classes matched across word frequency. These 30 pairs of items were then re-combined within each imageability class to provide the foil trials (e.g., throng-spade, agreement-threat).

Phonological processing was assessed via computerized administration of the Rhyme Judgment test from the PALPA (Test 15, Kay et al., 1992). This test consists of 60 pairs of written words, half of which rhyme and half of which do not. For the rhyming pairs, words can share both orthographic body and phonological rhyme (e.g., town-gown) or phonological rhyme only (e.g., horse-force). Similarly, the nonrhyming trials either differ on both dimensions (e.g., dome-bomb) or on phonological rhyme only (e.g., cheat-sweat).

##### Fluency tasks

In order to tap semantic and phonological processing in a format that did not require orthographic processing, participants were also given category and rhyme fluency tasks. These tasks are important because differences seen in tasks involving orthography may result directly from differences in reading style (i.e., processing of the input), whereas those seen on purely spoken tasks like fluency would indicate differences in the function of more primary language processing systems. Following Nation and Snowling (1998), the category fluency task asked people to generate: animals, ways of getting from one place to another, and kinds of work that people do. Similarly, the rhyme fluency task asked people to generate rhymes for: plate, fright, and chair.

#### Procedure

Participants completed all assessments in a single session in a fixed task order: word reading, nonword reading, synonym judgment, rhyme judgment, category fluency and rhyme fluency. All computerized tasks used DMDX software (Forster and Forster, 2003). The administration and scoring of the word and nonword reading tasks was identical to that in Experiment 1. For the synonym and rhyme judgment tasks, each trial involved presentation of a white fixation cross for 500 ms, followed by a written word pair in lower case until response, up to a maximum of 4000 ms. Responses were recorded using the shift keys on the keyboard, with the right shift key indicating a match decision and the left shift key indicating a mismatch decision. The next trial then appeared after 250 ms. For each task, participants completed six representative practice trials before completing the main block of 60 items, which were randomized anew for each participant. For the category and rhyme fluency tasks, participants were instructed to generate as many examples as possible within 1 min, and the prompt was spoken by the experimenter, with responses digitally recorded for later scoring.

### Results

As per Experiment 1, we computed inverse efficiency measures (Röder et al., 2007; Roberts et al., 2010) for each condition by dividing the correct reaction time with the proportion correct, then computed the consistency effect (inconsistent-consistent) for the low imageability items. These values act as a measure of semantic reliance during reading aloud, “SR index,” that is used as a continuous predictor in the regression analyses for all tasks.

#### Imageability

**Figure 3:**
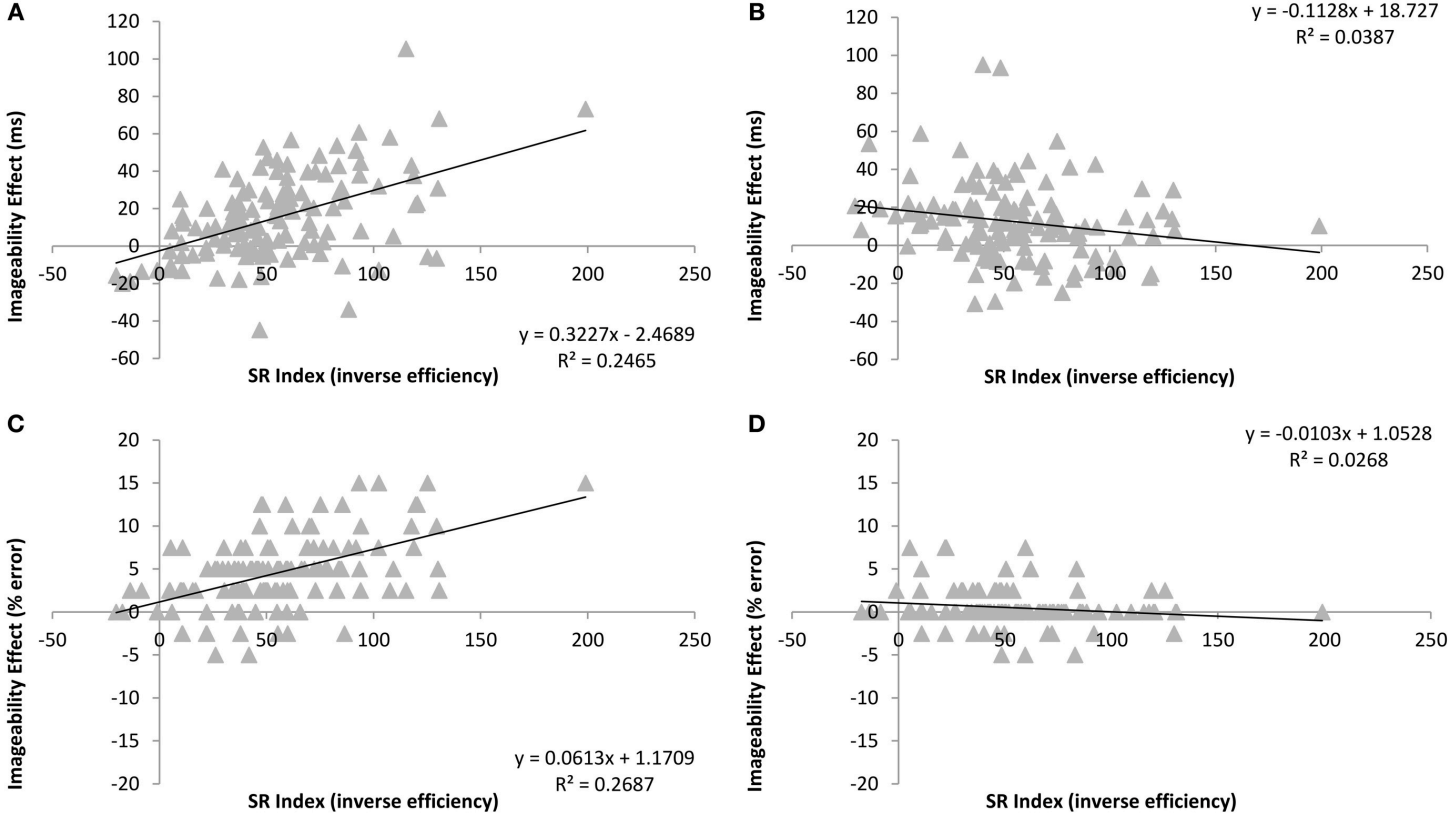
**Relationship between the degree of semantic reliance index (consistency effect for low imageability items in inverse efficiency scores) and imageability effects in word reading reaction times and accuracy according to consistency. (A,C)** show performance for the inconsistent words, and **(B,D)** show performance for consistent words.

Cell means and standard deviations for accuracy and error rates across all participants for the word reading task are provided in Table 8. In order to determine if the SR index uniquely predicted the size of the imageability effect for inconsistent words, it was included as a continuous predictor in repeated measures ANOVAs on RT and error rates with the within participant factors of imageability and consistency. There was a significant main effect of SR index [*F*_(1, 127)_ = 15.48, *p* < 0.0005; *F*_(1, 127)_ = 6.98, *p* = 0.009] due to the slower reaction times and higher error rates observed with increasing semantic reliance. There were significant main effects of consistency [*F*_(1, 127)_ = 4.11, *p* = 0.045; *F*_(1, 127)_ = 21.59, *p* < 0.0005] that were larger for readers with a higher SR index [*F*_(1, 127)_ = 20.99, *p* < 0.0005; *F*_(1, 127)_ = 66.88, *p* < 0.0005]. There were significant effects of imageability [*F*_(1, 127)_ = 10.78, *p* = 0.001; *F*_(1, 127)_ = 7.98, *p* = 0.006] that were larger for readers with a higher SR index [*F*_(1, 127)_ = 8.95, *p* = 0.003; *F*_(1, 127)_ = 22.42, *p* < 0.0005]. There was an interaction between consistency and imageability in RTs [*F*_(1, 127)_ = 24.09, *p* < 0.0005]. Most importantly, in both reaction times and error rates, there was a significant three-way interaction [*F*_(1, 127)_ = 43.72, *p* < 0.0005; *F*_(1, 127)_ = 64.50, *p* < 0.0005]. As can be seen in Figure 3, this interaction resulted from the positive relationship between the SR index and the size of the imageability effect for inconsistent words [*B* = 0.508, *t*_(128)_ = 6.64, *p* < 0.0005; *B* = 0.536, *t*_(128)_ = 7.16, *p* < 0.0005], as predicted. In addition, this relationship was reversed for the consistent words (*B* = −0.186, *t*_(128)_ = −2.13, *p* = 0.035; *B* = −0.160, *t*_(128)_ = −1.83, *p* = 0.070].

**Table 8:** **Average values (and standard deviations) of RT and error rates for the word reading task according to consistency and imageability**.

		**RT**	**Errors**
Consistent	High imageability	519 (69)	0.78 (1.76)
	Low imageability	532 (73)	1.2 (2.6)
Inconsistent	High imageability	531 (72)	3.26 (3.32)
	Low imageability	546 (79)	7.79 (4.12)

#### Nonwords

Nonword pronunciations were scored as per Experiment 1. Cell means and standard deviations are provided in Table 9. A repeated measures ANOVA on the percentage of extant body pronunciations for consistent and inconsistent nonwords that included SR index revealed only a marginally significant interaction [*F*_(1, 127)_ = 3.04, *p* = 0.084], which arose as greater semantic reliance showed a positive relationship with consistent nonwords and a negative relationship for inconsistent nonwords, but neither approached significance. A repeated measures ANOVA on the type of body pronunciation given for inconsistent nonwords that included SR index yielded only a main effect of pronunciation type [*F*_(1, 127)_ = 1517.62, *p* < 0.0005], as per Experiment 1. A repeated measures ANOVA on the consistent pronunciation RTs for consistent and inconsistent nonwords showed main effects of consistency [*F*_(1, 127)_ = 10.25, *p* = 0.002], and of SR index [*F*_(1, 127)_ = 8.62, *p* = 0.004], with increased semantic involvement corresponding to slower overall nonword reading, and no significant interaction, confirming the findings of Experiment 1.

**Table 9:** **Percentage extant body-rime pronunciations and reaction times for the most common body pronunciation according to consistency (standard deviations given in parentheses)**.

Consistent	% body pronunciation	94.2 (5.35)
	Consistent RT	613 (123)
Inconsistent	% body pronunciation	93.49 (5.13)
	Consistent RT	642 (123)
	Proportion 1	0.595 (0.054)
	Proportion 2	0.353 (0.048)
	Proportion 3	0.049 (0.021)
	Proportion 4	0.003 (0.006)

*For inconsistent words, the proportion of each type of body pronunciation is also provided*.

*Proportion 1 is that for the most common body-rime mapping, Proportion 4 is the least common body-rime mapping*.

#### Judgment tasks

Cell means and standard deviations for accuracy and error rates across all participants for the synonym judgment task are provided in Table 10. Repeated measures ANOVAs on RT and error rates with the within participant factors of decision type and imageability and SR index included as a continuous predictor revealed significant main effects of decision type [*F*_(1, 127)_ = 4.33, *p* = 0.039; *F*_(1, 127)_ = 53.09, *p* < 0.0005] and imageability [*F*_(1, 127)_ = 112.06, *p* < 0.0005; *F*_(1, 127)_ = 40.10, *p* < 0.0005], with an interaction between the two apparent in errors [*F*_(1, 112)_ = 13.57, *p* < 0.0005] due to the larger imageability effect seen for same than different trials. No effects involving SR index were significant [*F*s_(1, 127)_ < 2.24, *p*s > 0.137; *F*s_(1, 127)_ < 1.97, *ps* > 0.163].

**Table 10:** **Average values (and standard deviations) of RT and error rates for the synonym judgment task according to decision type and imageability**.

		**RT**	**Errors**
Same	High imageability	1014 (217)	10.59 (8.8)
	Low imageability	1263 (329)	23.26 (13.47)
Different	High imageability	1090 (253)	1.76 (4.21)
	Low imageability	1300 (295)	5.63 (8.35)

**Figure 4:**
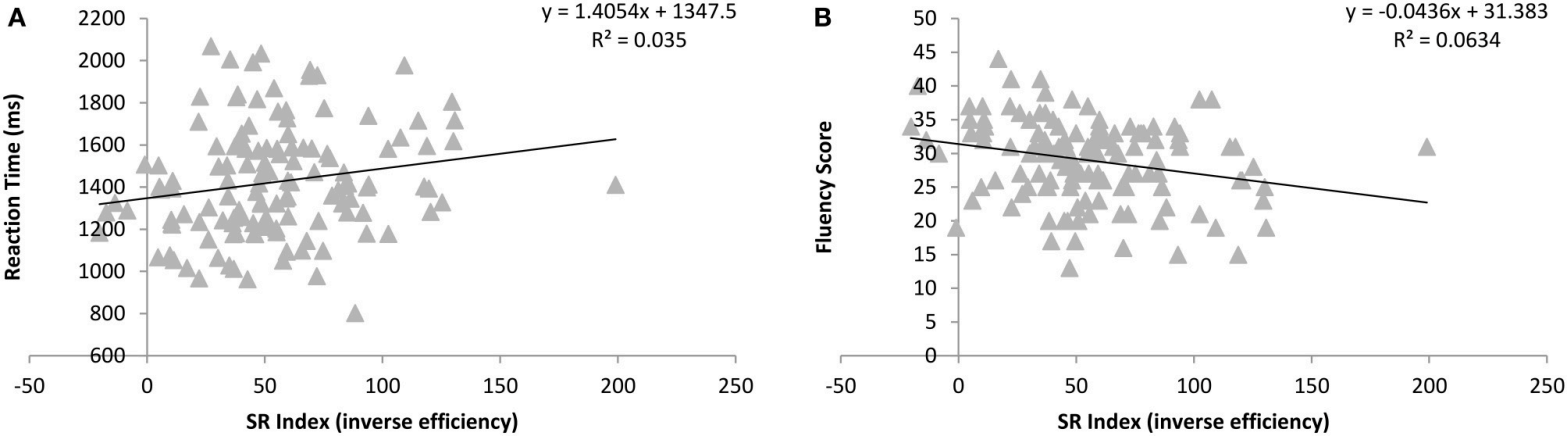
**Relationship between the degree of semantic reliance index (consistency effect for low imageability items in inverse efficiency scores) and overall reaction times in the rhyme judgment task (A) and number of correct responses in the rhyme fluency task (B)**.

Cell means and standard deviations for accuracy and error rates across all participants for the rhyme judgment task are provided in Table 11. Repeated measures ANOVAs on RT and error rates with the within participant factors of decision type and orthographic overlap SR index included as a continuous predictor revealed significant main effects of decision type [*F*_(1, 127)_ = 81.67, *p* < 0.0005; *F*_(1, 127)_ = 25.47, *p* < 0.0005]. This interacted with orthographic overlap [*F*_(1, 127)_ = 41.31, *p* < 0.0005; *F*_(1, 127)_ = 30.15, *p* < 0.0005], due to the fact that items with orthographic overlap were easier to accept as the same and harder to reject as different. As can be seen in Figure 4A, there was also a significant main effect of SR index, such that those with higher semantic reliance were slower [*F*_(1, 127)_ = 4.18, *p* = 0.043]. No other effects were significant [*F*s_(1, 127)_ < 0.49, *p*s > 0.487; *F*s_(1, 127)_ < 1.28, *p*s > 0.261].

**Table 11:** **Average values (and standard deviations) of RT and error rates for the rhyme judgment task according to decision type and overlap type**.

		**RT**	**Errors**
Same	Orthographic	1183 (250)	5.17 (6.52)
	Phonological	1344 (278)	15.35 (13.5)
Different	Orthographic	1686 (373)	31.01 (19.82)
	Phonological	1485 (296)	15.92 (12.84)

#### Fluency

Overall, there were an average of 56 (*SD* = 12) correct exemplars produced within the time limit in the category fluency task. A regression using SR index to predict semantic fluency score did not reveal a significant relationship [*B* = −0.107, *t*_(128)_ = −1.21, *p* = 0.225]. Overall, there were an average of 29 (*SD* = 6) correct rhymes produced within the time limit in the rhyme fluency task. A regression using SR index to predict rhyme fluency score did reveal a significant negative relationship [*B* = −0.258, *t*_(128)_ = −2.58, *p* = 0.003], such that those with a stronger semantic involvement produced fewer correct rhymes, as can be seen in Figure 4B.

### Discussion

The results of Experiment 2 confirm and extend those of Experiment 1 in a number of ways. Firstly, the relationship between our proposed index of semantic reliance (i.e., the size of the consistency effect for low imageability words in inverse efficiency) and the size of the imageability effect specifically for inconsistent words was validated in a large scale regression design. Secondly, as per Experiment 1, there were no systematic differences in the nature of inconsistent nonword pronunciations or the size of the consistency effect in RTs according to degree of semantic involvement. In other words, even with this more powerful approach, there was no evidence for the direct route specialization that might be expected according to the division of labor hypothesis. Indeed, the trend toward slower nonword reading for high SR readers seen in Experiment 1 was confirmed here by a significant positive relationship between degree of semantic reliance and nonword reading RTs. Hence it seems that increased semantic reliance is associated with less efficient direct route processing.

In order to determine if variation in the SR index is associated with individual differences in language processing more generally, as might be expected according to the primary systems view (Patterson and Lambon Ralph, 1999), we considered performance on tasks tapping semantic and phonological processing capacity. The SR index did not reliably predict performance when the task was to judge whether pairs of written words were synonyms, but higher semantic reliance was associated with slower RTs to judge whether pairs of written words rhyme. Similarly, although the SR index was not related to ability to generate exemplars to spoken category names, higher semantic reliance was associated with less success in generating rhymes to spoken words. Overall then, these additional tasks demonstrate that (a) the SR index does not tap a general cognitive capacity as it is not related to performance across all tasks; (b) degree of semantic reliance in reading is not related to general semantic processing capacity; and (c) increased semantic reliance during reading aloud is related to poorer performance on phonological processing tasks with both written and spoken stimuli. This last result agrees with the evidence from nonword reading indicating less efficient direct route processing for participants with higher semantic reliance, and is in line with the known relationship between nonword reading and phonological processing (Pennington et al., 1990).

## General discussion

This study aimed to establish the cognitive correlates of individual differences in semantic reliance during exception word reading, operationalized as the size of the consistency effect seen for low imageability words. The connectionist triangle model proposes that individual differences in the size of semantic effects should be seen specifically for words with atypical spelling-sound correspondences. In Experiment 1 we sub-grouped participants according to the SR index, and as predicted, the high SR readers showed a significantly larger imageability effect than low SR readers for inconsistent words in errors, but no such difference was apparent for consistent words. It might perhaps be argued that this could reflect some difference in terms of vocabulary knowledge; but in a semantic priming task, the high SR readers showed a significantly larger priming effect than low SR readers in RTs specifically for inconsistent words. In Experiment 2, a regression approach was used to further assess differences in degree of semantic reliance according to our SR index: here, increasing semantic involvement corresponded to larger imageability effects in inconsistent but not consistent words in both RTs and errors.

Overall then, it would seem that the size of the consistency effect for low imageability words provides a good measure of degree of semantic reliance during reading aloud and, as predicted by the connectionist triangle model, there are systematic and considerable individual differences between normal readers along this dimension. The specificity of these effects to the inconsistent words shows that this is not a global semantic reading strategy, but rather a graded effect most apparent for items with atypical mappings between spelling and sound. Furthermore, performance on a synonym judgment task and a category fluency task bore no significant relationship to the SR index. This demonstrates that the higher semantic reliance seen during reading aloud is task specific, such that the SR index is not tapping a more general processing capacity, such as vocabulary or intelligence.

While the present results certainly support the connectionist triangle model predictions of principled individual differences in terms of the degree of semantic reliance during reading aloud for spelling-sound atypical items, there was little evidence for concomitant differences in specialization of the direct pathway. According to the division of labor hypothesis, when trained in the presence of semantic support, the direct pathway gradually comes to specialize in common and consistent mappings between spelling and sound. Hence, when the operation of the direct pathway is laid bare via nonword reading, those individuals showing a high degree of semantic reliance during word reading might be expected to produce pronunciations of inconsistent nonwords according to the most common mappings and to do so relatively quickly given the lack of competition from alternative mappings. Such systematic differences in terms of the pronunciations given to nonwords or the size of the consistency effect according to degree of semantic reliance were not apparent in either experiment.

What we did observe, rather, was slower overall nonword reading RTs with increasing semantic involvement. Further considerations of phonological processing abilities in Experiment 2 revealed that those readers with higher semantic involvement during word reading were slower (although no less accurate) to judge whether written words rhyme, and generated fewer rhymes to a spoken prompt in a limited time period. These results are in accordance with those of Strain and Herdman (1999), who classified readers according to their phonological processing skill, as measured by a combination of nonword reading and phoneme blending ability. Readers of lower phonological skill showed a much larger regularity effect for low imageability items and a much larger imageability effect for exception items in error rates relative to the high phonological skill readers.

Overall, our results suggest that rather than being associated with increased direct pathway efficiency, greater semantic reliance during reading aloud is associated with less well developed phonological representations, at least at the level of the rhyme. We speculate that one possible explanation for this pattern of results is that a mild disadvantage in phonological processing might in fact have provided the impetus for increased reliance on semantic activation to cope with words with atypical spelling-sound mappings in the first instance, as these load highly on phonological processing due to the conflicting pronunciations they activate (Woollams and Patterson, 2012).

In simulations of developmental dyslexia, Harm and Seidenberg (1999) explored the consequences of impairing the phonological representations of a connectionist model of the direct pathway before training it to read. They found that while this impaired nonword reading accuracy, it also impacted upon exception word reading ability, consistent with the mixed pattern often seen in developmental phonological dyslexia (Manis et al., 1996, cf. Castles and Coltheart, 1993; McDougall et al., 2005), which is accompanied by deficits in phonological tasks like phoneme position analysis. As the model did not include an implementation of the semantic system, the effects of this pre-reading phonological damage on the division of labor are unknown; but it seems plausible that one way in which that model could offset these difficulties in exception word reading would be to increase semantic reliance. By such an account, the degree of semantic reliance is determined by the competence of the direct pathway (as explored in the simulations of Plaut (1997) using weight decay manipulations), which is in turn determined by the pre-reading state of the phonological system (as shown by the simulations of Harm and Seidenberg (1999) described above). Hence normal readers with stronger semantic reliance will show slower nonword reading performance and mild but measurable residual difficulties in phonological tasks, as observed here.

The finding of poorer direct pathway function and phonological processing weaknesses for readers with higher semantic reliance for exception word reading are highly consistent with the neural correlates of the SR index, as revealed by functional imaging. In line with the division of labor hypothesis (Plaut et al., 1996) and the prevalence of surface dyslexia in semantic dementia (Woollams et al., 2007), Hoffman et al. (2015) found that a semantic region of interest in the left lateral anterior temporal lobe showed higher activation during reading of exception as opposed to regular words. Moreover, in accordance with the idea that there are individual differences in the degree of semantic reliance during reading (Plaut, 1997; Woollams et al., 2007), this same area showed a strong positive correlation with the SR index. This result is consistent with both the association of the whole-word reading pathway with semantic processing in the anterior and inferior temporal regions in functional imaging meta-analyses (Cattinelli et al., 2013), and the correlation of left anterior temporal damage with exception word reading deficits (Brambati et al., 2009). It also agrees with structural neuroimaging results demonstrating that exception word reading abilities in normal participants correlates with cortical thickness in bilateral anterior temporal regions (Blackmon et al., 2010).

Of particular interest, given our finding of poorer nonword reading and spoken and written rhyme processing amongst high SR readers, is that Hoffman et al. (2015) also observed that a phonological region of interest in the left pre-central gyrus was more active during reading of regular as opposed to exception words, implicating it in the direct pathway, and it was this same region that showed a significant negative correlation with SR index. In other words, the higher the semantic reliance, the lower the activation in regions associated with the direct orthography to phonology processing. Indeed, damage to this area has been associated with deficits in both nonword reading and phonological processing in primary progressive aphasia (Henry et al., 2012). It has also been associated with phonological processing in functional meta-analyses (Vigneau et al., 2006), hence we would expect it to be involved in written and spoken rhyme processing. The focus of lower activations for higher semantic reliance individuals in this part of the direct pathway therefore supports the possibility that the inefficient function of this pathway may have its origins in phonological capacity. The neuroanatomical basis for these individual differences in nonword reading and rhyme processing remain unknown, and need to be explored in future research.

## Conclusion

Current computational models of reading aloud vary considerably in the importance they ascribe to semantic activation to support reading of words with exceptional spelling-sound correspondences. Individual differences in the degree of semantic reliance during exception word reading are of particular theoretical importance as they have been offered by the connectionist triangle model as an account of variation in the degree of surface dyslexia observed in semantic dementia patients with degraded conceptual knowledge. This account has been supported by the correlation of a behavioral index of the degree of semantic reliance for exception word reading with activation in left anterior temporal regions associated with semantic processing. Our study has demonstrated that high semantic reliance readers also show a stronger impact of both imageability and semantic priming upon reading, but specifically for exception words, as predicted by the connectionist triangle model account. Moreover, our study has revealed for the first time that higher semantic reliance readers show inefficient processing via the direct pathway in terms of speed of nonword reading and also less efficient phonological processing, as measured by written and spoken rhyme processing tasks. These latter findings concur with recent functional imaging data showing lesser activation in higher semantic reliance readers in left pre-central gyrus, which is associated with phonological processing.

Our results are compatible with a proposal that individual differences in the degree of semantic reliance for exception word reading stem from initial phonological processing weaknesses that undermine the development of an efficient direct pathway, which is then compensated for by the semantic pathway. This hypothesis could be assessed by future simulations in large scale connectionist computational models of reading aloud that incorporate semantic representations and a developmentally realistic training regime. Within this framework, the pre-reading phonological representations in the model could be impaired and the impact on the subsequent degree of semantic reliance for exception words could be examined. Simulations could also be conducted to explore the underlying causes of differences in phonological representations in a number of ways, including manipulating aspects of training of the mappings between semantics and phonology to emulate environmental contributions and varying structural dimensions of the model, such as the number of hidden units or degree of interconnection, to approximate the neural level.

## Author contributions

AW, MLR, and KP designed the study; AW and GM collected and analyzed the data; AW, MLR, and KP wrote the paper.

### Conflict of interest statement

The authors declare that the research was conducted in the absence of any commercial or financial relationships that could be construed as a potential conflict of interest.

## References

[B1] Andrews S. Scarratt D. R. (1998). Rule and analogy mechanisms in reading non words: hough dou peapel rede gnew wirds? J. Exp. Psychol. Hum. Percept. Perform. 24, 1052–1086. 10.1037/0096-1523.24.4.1052

[B2] Baayen R. H. Piepenbrock R. Van Rijn H. (1993). The CELEX Lexical Database (CD-ROM). Philadelphia, PA: Linguistic Data Consortium, University of Pennsylvania.

[B3] Balota D. A. Cortese M. J. Sergent-Marshall S. D. Spieler D. H. Yap M. J. (2004). Visual word recognition of single-syllable words. J. Exp. Psychol. Gen. 133, 283–316. 10.1037/0096-3445.133.2.283 15149254

[B4] Baron J. Strawson C. (1976). Use of orthographic and word-specific knowledge in reading words aloud. J. Exp. Psychol. Hum. Percepti. Perform. 2, 386–393. 10.1037/0096-1523.2.3.386

[B5] Blackmon K. Barr W. B. Kuzniecky R. Dubois J. Carlson C. Quinn B. T.. (2010). Phonetically irregular word pronunciation and cortical thickness in the adult brain. Neuroimage 51, 1453–1458. 10.1016/j.neuroimage.2010.03.028 20302944 PMC2873116

[B6] Blazely A. M. Coltheart M. Casey B. J. (2005). Semantic impairment with and without surface dyslexia: implications for models of reading. Cogn. Neuropsychol. 22, 695–717. 10.1080/02643290442000257 21038273

[B7] Brambati S. M. Ogar J. Neuhaus J. Miller B. L. Gorno-Tempini M. L. (2009). Reading disorders in primary progressive aphasia: a behavioral and neuroimaging study. Neuropsychologia 47, 1893–1900. 10.1016/j.neuropsychologia.2009.02.033 19428421 PMC2734967

[B8] Brown P. Lupker S. J. Colombo L. (1994). Interacting sources of information in word naming: a study of individual differences. J. Exp. Psychol. Hum. Percept. Perform. 20, 537–554. 10.1037/0096-1523.20.3.537

[B9] Brysbaert M. New B. (2009). Moving beyond Kučera and Francis: a critical evaluation of current word frequency norms and the introduction of a new and improved word frequency measure for American English. Behav. Res. Methods 41, 977–990. 10.3758/BRM.41.4.977 19897807

[B10] Brysbaert M. Van Wijnendaele I. De Deyne S. (2000). Age-of-acquisition effects in semantic processing tasks. Acta Psychol. (Amst). 104, 215–226. 10.1016/S0001-6918(00)00021-4 10900706

[B11] Castles A. Coltheart M. (1993). Varieties of developmental dyslexia. Cognition 47, 149–180. 10.1016/0010-0277(93)90003-E 8324999

[B12] Cattinelli I. Borghese N. A. Gallucci M. Paulesu E. (2013). Reading the reading brain: A new meta-analysis of functional imaging data on reading. J. Neurolinguistics 26, 214–238. 10.1016/j.jneuroling.2012.08.001

[B13] Cipolotti L. Warrington E. K. (1995). Semantic memory and reading abilities: a case report. J. Int. Neuropsychol. Soc. 1, 104–110. 10.1017/S1355617700000163 9375215

[B14] Coltheart M. Rastle K. Perry C. Langdon R. Ziegler J. (2001). DRC: a dual route cascaded model of visual word recognition and reading aloud. Psychol. Rev. 108, 204–256. 10.1037/0033-295X.108.1.204 11212628

[B15] Coltheart M. Tree J. J. Saunders S. J. (2010). computational modeling of reading in semantic dementia: comment on woollams, lambon ralph, plaut, and patterson (2007). Psychol. Rev. 117, 256–271. 10.1037/a0015948 20063972

[B16] Cortese M. J. Fugett A. (2004). Imageability ratings for 3,000 monosyllabic words. Behav. Res. Methods Instrum. Comput. 36, 384–387. 10.3758/BF03195585 15641427

[B17] Cortese M. J. Simpson G. B. (2000). Regularity effects in word naming: what are they? Mem. Cogn. 28, 1269–1276. 10.3758/BF03211827 11219954

[B18] Cortese M. J. Simpson G. B. Woolsey S. (1997). Effects of association and imageability on phonological mapping. Psychon. Bull. Rev. 4, 226–231. 10.3758/BF03209397 21331829

[B19] Dilkina K. McClelland J. L. Plaut D. C. (2008). A single-system account of semantic and lexical deficits in five semantic dementia patients. Cogn. Neuropsychol. 25, 136–164. 10.1080/02643290701723948 18568816

[B20] Forster K. I. Forster J. C. (2003). DMDX: a Windows display program with millisecond accuracy. Behav. Res. Methods Instrum. Comput. 35, 116–124. 10.3758/BF03195503 12723786

[B21] Fraser J. Goswami U. Conti-Ramsden G. (2010). Dyslexia and specific language impairment: the role of phonology and auditory processing. Sci. Studi. Read. 14, 8–29. 10.1080/10888430903242068

[B22] Graham N. L. Patterson K. Hodges J. R. (2000). The impact of semantic memory impairment on spelling: evidence from semantic dementia. Neuropsychologia 38, 143–163. 10.1016/S0028-3932(99)00060-3 10660226

[B23] Harm M. W. Seidenberg M. S. (1999). Phonology, reading acquisition, and dyslexia: insights from connectionist models. Psychol. Rev. 106, 491–528. 10.1037/0033-295X.106.3.491 10467896

[B24] Harm M. W. Seidenberg M. S. (2004). Computing the meanings of words in reading: cooperative division of labor between visual and phonological processes. Psychol. Rev. 111, 662–720. 10.1037/0033-295X.111.3.662 15250780

[B25] Henry M. L. Beeson P. M. Alexander G. E. Rapcsak S. Z. (2012). Written language impairments in primary progressive aphasia: a reflection of damage to central semantic and phonological processes. J. Cogn. Neurosci. 24, 261–275. 10.1162/jocn_a_00153 22004048 PMC3307525

[B26] Hoeft F. Hernandez A. McMillon G. Taylor-Hill H. Martindale J. L. Meyler A.. (2006). Neural basis of dyslexia: a comparison between dyslexic and nondyslexic children equated for reading ability. J. Neurosci. 26, 10700–10708. 10.1523/JNEUROSCI.4931-05.2006 17050709 PMC6674758

[B27] Hoffman P. Lambon Ralph M. A. Woollams A. M. (2015). Triangulation of the neurocomputational architecture underpinning reading aloud. Proc. Natl. Acad. Sci. U.S.A. 112, E3719–E3728. 10.1073/pnas.1502032112 26124121 PMC4507229

[B28] Jared D. (1997). Spelling-sound consistency affect the naming of high-frequency words. J. Mem. Lang. 36, 505–529. 10.1006/jmla.1997.2496

[B29] Jared D. (2002). Spelling-sound consistency and regularity effects in word naming. J. Mem. Lang. 46, 723–750. 10.1006/jmla.2001.2827

[B30] Jefferies E. Rogers T. T. Ralph M. A. L. (2011). Premorbid expertise produces category-specific impairment in a domain-general semantic disorder. Neuropsychologia 49, 3213–3223. 10.1016/j.neuropsychologia.2011.07.024 21816166 PMC3192291

[B31] Kay J. Lesser R. Coltheart M. (1992). Psycholinguistic Assessments of Language Processing in Aphasia (PALPA). Hove: Lawrence Erlbaum Associates.

[B32] Kucera H. Francis W. N. (1967). Computational Analysis of Present-Day American English. Providence, RI: Brown University Press.

[B33] Kuperman V. Stadthagen-Gonzalez H. Brysbaert M. (2012). Age-of-acquisition ratings for 30,000 English words. Behav. Res. Methods 44, 978–990. 10.3758/s13428-012-0210-4 22581493

[B34] Lambon Ralph M. A. Ehsan S. (2006). Age of acquisition effects depend on the mapping between representations and the frequency of occurrence: empirical and computational evidence. Vis. Cogn. 13, 928–948. 10.1080/13506280544000110

[B35] Maki W. S. Krimsky M. Muñoz S. (2006). An efficient method for estimating semantic similarity based on feature overlap: reliability and validity of semantic feature ratings. Behav. Res. Methods 38, 153–157. 10.3758/BF03192761 16817525

[B36] Manis F. R. Seidenberg M. S. Doi L. M. McBride-Chang C. Petersen A. (1996). On the bases of two subtypes of development dyslexia. Cognition 58, 157–195. 10.1016/0010-0277(95)00679-6 8820386

[B37] McDougall P. Borowsky R. Mackinnon G. E. Hymel S. (2005). Process dissociation of sight vocabulary and phonetic decoding in reading: a new perspective on surface and phonological dyslexias. Brain Lang. 92, 185–203. 10.1016/j.bandl.2004.06.003 15629491

[B38] McFalls E. L. Schwanenflugel P. J. Stahl S. A. (1996). Influence of word meaning on the acquisition of a reading vocabulary in second-grade children. Read. Writ. 8, 235–250. 10.1007/BF00420277

[B39] Nation K. Snowling M. J. (1998). Semantic processing and the development of word-recognition skills: evidence from children with reading comprehension difficulties. J. Mem. Lang. 39, 85–101. 10.1006/jmla.1998.2564

[B40] Patterson K. Hodges J. R. (1992). Deterioration of word meaning: implications for reading. Neuropsychologia 30, 1025–1040. 10.1016/0028-3932(92)90096-5 1484600

[B41] Patterson K. Lambon Ralph M. A. (1999). Selective disorders of reading? Curr. Opin. Neurobiol. 9, 235–239. 10.1016/S0959-4388(99)80033-6 10322178

[B42] Patterson K. Marcel A. (1992). Phonological ALEXIA or PHONOLOGICAL alexia? in Analytic Approaches to Human Cognition, eds Alegria J. Holdender J. Junca De Morais J. Radean M. (Amsterdam: Elsevier Science), 259–274.

[B43] Patterson K. Plaut D. C. McClelland J. L. Seidenberg M. S. Behrmann M. Hodges J. R. (1996). Connections and disconnections: a connectionist account of surface dyslexia, in Neural Modelling of Cognitive and Brain Disorders, ed Ruppin E. (Singapore: World Scientific), 177–199.

[B44] Paivio A. Yuille J. C. Madigan S. A. (1968). Concreteness, imagery, and meaningfulness values for 925 nouns. J. Exp. Psychol. 76(Pt. 2), 1–25. 10.1037/h0025327 5672258

[B45] Pennington B. F. Van Orden G. C. Smith S. D. Green P. A. Haith M. M. (1990). Phonological processing skills and deficits in adult dyslexics. Child Dev. 61, 1753–1778. 10.2307/1130836 2083497

[B46] Plaut D. C. (1997). Structure and function in the lexical system: insights from distributed models of word reading and lexical decision. Lang. Cogn. Process. 12, 765–805. 10.1080/016909697386682

[B47] Plaut D. C. Booth J. R. (2000). Individual and developmental differences in semantic priming: empirical and computational support for a single-mechanism account of lexical processing. Psychol. Rev. 107, 786–823. 10.1037/0033-295X.107.4.786 11089407

[B48] Plaut D. C. McClelland J. L. Seidenberg M. S. Patterson K. (1996). Understanding normal and impaired word reading: computational principles in quasi-regular domains. Psychol. Rev. 103, 56–115. 10.1037/0033-295X.103.1.56 8650300

[B49] Plaut D. C. Shallice T. (1993). Deep dyslexia: a case study of cormectionist neuropsychology. Cogn. Neuropsychol. 10, 377–500. 10.1080/02643299308253469

[B50] Roberts D. J. Lambon Ralph M. A. Woollams A. M. (2010). When does less yield more? The impact of severity upon implicit recognition in pure alexia. Neuropsychologia 48, 2437–2446. 10.1016/j.neuropsychologia.2010.04.002 20406652

[B51] Röder B. Kusmierek A. Spence C. Schicke T. (2007). Developmental vision determines the reference frame for the multisensory control of action. Proc. Natl. Acad. Sci. U.S.A. 104, 4753–4758. 10.1073/pnas.0607158104 17360596 PMC1838672

[B52] Seidenberg M. S. McClelland J. L. (1989). A distributed, developmental model of word recognition and naming. Psychol. Rev. 96, 523–568. 10.1037/0033-295X.96.4.523 2798649

[B53] Shallice T. (1988). From Neuropsychology to Mental Structure. Cambridge: Cambridge University Press.

[B54] Shibahara N. Zorzi M. Hill M. P. Wydell T. Butterworth B. (2003). Semantic effects in word naming: evidence from English and Japanese Kanji. Q. J. Exp. Psychol. A Hum. Exp. Psychol. 56A, 263–286. 10.1080/02724980244000369 12613564

[B55] Strain E. Herdman C. M. (1999). Imageability effects in word naming: an individual differences analysis. Can. J. Exp. Psychol. 53, 347–359. 10.1037/h0087322 10646206

[B56] Strain E. Patterson K. Seidenberg M. S. (1995). Semantic effects in single-word naming. J. Exp. Psychol. Learn. Mem. Cogn. 21, 1140–1154. 10.1037/0278-7393.21.5.1140 8744959

[B57] Veneszky R. L. (1970). The Structure of English Orthography. The Hague: Mouton.

[B58] Vigneau M. Beaucousin V. Hervé P. Y. Duffau H. Crivello F. Houdé O.. (2006). Meta-analyzing left hemisphere language areas: phonology, semantics, and sentence processing. Neuroimage 30, 1414–1432. 10.1016/j.neuroimage.2005.11.002 16413796

[B59] Woollams A. M. (2005). Imageability and ambiguity effects in speeded naming: convergence and divergence. J. Exp. Psychol. Learn. Mem. Cogn. 31, 878–890. 10.1037/0278-7393.31.5.878 16248739

[B60] Woollams A. M. Patterson K. (2012). The consequences of progressive phonological impairment for reading aloud. Neuropsychologia 50, 3469–3477. 10.1016/j.neuropsychologia.2012.09.020 23000132

[B61] Woollams A. M. Ralph M. A. L. Plaut D. C. Patterson K. (2007). SD-squared: on the association between semantic dementia and surface dyslexia. Psychol. Rev. 114, 316–339. 10.1037/0033-295X.114.2.316 17500629

[B62] Ziegler J. C. Stone G. O. Jacobs A. M. (1997). What is the pronunciation for *-ough* and the spelling for /u/? A database for computing feedforward and feedback consistency in English. Behav. Res. Methods Instr. Comput. 29, 600–618. 10.3758/BF03210615

