# Supplementary material for: Do You Read How I Read? Systematic Individual Differences in Semantic Reliance amongst Normal Readers
Source: Front Psychol. 2016 Nov 22;7:1757. doi: 10.3389/fpsyg.2016.01757 (PMC5118465; doi:10.3389/fpsyg.2016.01757)
Supplement: Supplementary file 1 [file DataSheet1.docx]

Appendix A:

Imageability Stimuli

| Consistent | | Inconsistent | |
| --- | --- | --- | --- |
| High Imageability | Low Imageability | High Imageability | Low Imageability |
| bird | bide | bush | base |
| branch | bribe | breast | break |
| brick | bring | broad | brood |
| keg | curse | key | cache |
| cape | came | case | caste |
| chain | chill | cheese | choose |
| cloud | cope | class | cost |
| dawn | dumb | down | done |
| pole | pop | post | put |
| floor | flair | flood | flow |
| fresh | frock | frost | front |
| foil | fill | foul | full |
| gun | gape | gas | gone |
| globe | greed | glove | grow |
| hedge | hence | height | halve |
| line | large | limb | lease |
| life | luck | love | lose |
| goat | gain | geese | give |
| moon | meek | monk | mood |
| meal | mirth | mouth | mould |
| fudge | fraud | foot | phase |
| frill | frank | friend | phrase |
| park | place | pint | plait |
| pipe | pledge | pear | plead |
| point | price | pass | prow |
| pill | pomp | poll | pull |
| pale | poor | path | push |
| room | wrest | rough | wrath |
| sob | sense | son | salve |
| sky | scorn | ski | scarce |
| ship | sheer | shoe | show |
| sneer | slang | snow | slow |
| sock | soon | soot | soul |
| spoon | spite | sphere | spook |
| steam | stale | steak | stood |
| sweep | swell | sweat | swear |
| throat | think | throw | threat |
| tile | tame | town | tow |
| vine | vile | vase | vow |
| week | wish | wood | wear |

Appendix B

Priming Stimuli

| Set A | | Set B | |
| --- | --- | --- | --- |
| Prime | Target | Prime | Target |
| Consistent | | | |
| card | ace | ale | beer |
| chime | bell | fault | blame |
| cycle | bike | bang | boom |
| shore | beach | mind | brain |
| spawn | breed | hint | clue |
| pier | bridge | penny | coin |
| hum | buzz | theft | crime |
| hook | claw | trench | ditch |
| alarm | clock | fawn | deer |
| milk | cream | play | game |
| scratch | dent | purpose | goal |
| glory | fame | silver | gold |
| paste | glue | remorse | guilt |
| faith | hope | raisin | grape |
| jump | leap | wander | roam |
| parade | march | pouch | sack |
| search | seek | dessert | sweet |
| summit | top | squad | team |
| cry | weep | spin | wheel |
| income | wealth | bleach | white |
| Inconsistent | | | |
| pain | ache | shower | bath |
| necklace | bead | heart | blood |
| cub | bear | blast | bomb |
| whiskers | beard | trumpet | brass |
| soup | broth | fool | clown |
| tan | brown | hair | comb |
| cattle | bull | fold | crease |
| ascend | climb | murder | death |
| fabric | cloth | bread | dough |
| jewel | crown | meter | gauge |
| toy | doll | haunt | ghost |
| dish | food | goblin | ghoul |
| crystal | glass | robe | gown |
| guest | host | pasture | grass |
| joke | laugh | lipstick | rouge |
| shift | move | stitch | sew |
| north | south | marsh | swamp |
| grave | tomb | feel | touch |
| battle | war | rinse | wash |
| hornet | wasp | value | worth |

Appendix C

Nonword Stimuli

| Consistent | | Inconsistent | |
| --- | --- | --- | --- |
| lird | plide | fush | pase |
| canch | pibe | meast | heak |
| swick | pling | poad | slood |
| feg | lurse | vey | tache |
| lape | hame | hase | plaste |
| flain | shill | spheese | phoose |
| gloud | swope | stass | wost |
| frawn | fumb | cown | pone |
| spole | gop | rost | lut |
| voor | sair | frood | scow |
| wesh | wock | shost | lont |
| poil | prill | poul | sull |
| mun | bape | las | vone |
| sobe | cheed | brove | fow |
| cledge | wrence | deight | malve |
| bine | carge | pimb | mease |
| tife | guck | pove | bose |
| poat | dain | meese | sive |
| poon | veek | sonk | cood |
| breal | pirth | bouth | gould |
| pudge | taud | poot | shase |
| rill | brank | briend | thrase |
| sark | frace | sint | wrait |
| fripe | bredge | kear | pead |
| goint | pice | fass | frow |
| brill | fromp | froll | brull |
| thrale | coor | gath | cush |
| shoom | mest | wough | grath |
| pob | mense | lon | chalve |
| sny | smorn | sni | carce |
| stip | theer | choe | dow |
| skeer | cug | clow | swow |
| gock | froon | goot | floul |
| foon | grite | swere | stook |
| peam | spale | fleak | plood |
| meep | stell | gleat | prear |
| choat | flink | skow | phreat |
| kile | scame | pown | spow |
| hine | sile | tase | gow |
| deek | lish | throod | brear |

Appendix D

Scheme used for classification of nonword pronunciations. Pronunciations are ranked according to a token measure computed over all monosyllables in the CELEX corpus, and ties on this type measure are broken by summed frequency of the tokens for that pronunciation (per million).

| Nonword | PRON1 | SumCEL | tokens | PRON2 | SumCEL | tokens | PRON3 | SumCEL | tokens | PRON4 | SumCEL | tokens |
| --- | --- | --- | --- | --- | --- | --- | --- | --- | --- | --- | --- | --- |
| pase | base | 431.5084 | 3 | phase | 60.44693 | 2 | vase | 3.96648 | 1 |  | 0 | 0 |
| heak | beak | 221.3966 | 13 | break | 105.2514 | 2 |  | 0 | 0 |  | 0 | 0 |
| slood | brood | 295.8659 | 5 | good | 1153.24 | 4 | blood | 154.7486 | 2 |  | 0 | 0 |
| tache | ache | 4.748603 | 1 | cache | 0.837989 | 1 |  | 0 | 0 |  | 0 | 0 |
| plaste | waste | 129.8883 | 6 | caste | 4.804469 | 1 |  | 0 | 0 |  | 0 | 0 |
| phoose | goose | 49.77654 | 4 | choose | 59.21788 | 1 |  | 0 | 0 |  | 0 | 0 |
| wost | most | 1229.609 | 4 | cost | 345.3073 | 3 | dost | 0.614525 | 1 |  | 0 | 0 |
| pone | bone | 272.2905 | 13 | done | 3574.19 | 3 | shone | 249.8324 | 3 |  | 0 | 0 |
| lut | but | 5196.201 | 14 | put | 619.1061 | 1 |  | 0 | 0 |  | 0 | 0 |
| scow | brow | 2754.358 | 14 | blow | 1795.084 | 14 |  | 0 | 0 |  | 0 | 0 |
| lont | front | 249.162 | 2 | font | 1.340782 | 1 |  | 0 | 0 |  | 0 | 0 |
| sull | cull | 62.23464 | 9 | bull | 350.7263 | 3 |  | 0 | 0 |  | 0 | 0 |
| vone | bone | 272.2905 | 13 | done | 3574.19 | 3 | shone | 249.8324 | 3 |  | 0 | 0 |
| fow | brow | 2754.358 | 14 | blow | 1795.084 | 14 |  | 0 | 0 |  | 0 | 0 |
| malve | valve | 4.357542 | 2 | calve | 1.564246 | 2 |  | 0 | 0 |  | 0 | 0 |
| mease | please | 152.4022 | 4 | lease | 17.43017 | 3 |  | 0 | 0 |  | 0 | 0 |
| bose | hose | 1235.587 | 8 | lose | 291.3966 | 2 | dose | 6.759777 | 1 |  | 0 | 0 |
| sive | dive | 364.3575 | 11 | give | 653.1844 | 2 |  | 0 | 0 |  | 0 | 0 |
| cood | brood | 295.8659 | 5 | good | 1153.24 | 4 | blood | 154.7486 | 2 |  | 0 | 0 |
| gould | could | 5424.469 | 3 | mould | 11 | 1 |  | 0 | 0 |  | 0 | 0 |
| shase | base | 431.5084 | 3 | phase | 60.44693 | 2 | vase | 3.96648 | 1 |  | 0 | 0 |
| thrase | base | 431.5084 | 3 | phase | 60.44693 | 2 | vase | 3.96648 | 1 |  | 0 | 0 |
| wrait | bait | 152.1229 | 5 | plait | 1.787709 | 1 |  | 0 | 0 |  | 0 | 0 |
| pead | dead | 809.8883 | 8 | bead | 394.5251 | 6 |  | 0 | 0 |  | 0 | 0 |
| frow | brow | 2754.358 | 14 | blow | 1795.084 | 14 |  | 0 | 0 |  | 0 | 0 |
| brull | cull | 62.23464 | 9 | bull | 350.7263 | 3 |  | 0 | 0 |  | 0 | 0 |
| cush | gush | 81.73184 | 13 | bush | 84.13408 | 3 |  | 0 | 0 |  | 0 | 0 |
| grath | bath | 101.3966 | 3 | math | 5.921788 | 3 | wrath | 7.039106 | 1 |  | 0 | 0 |
| chalve | valve | 4.357542 | 2 | calve | 1.564246 | 2 |  | 0 | 0 |  | 0 | 0 |
| carce | scarce | 9.106145 | 1 | farce | 3.463687 | 1 |  | 0 | 0 |  | 0 | 0 |
| dow | brow | 2754.358 | 14 | blow | 1795.084 | 14 |  | 0 | 0 |  | 0 | 0 |
| swow | brow | 2754.358 | 14 | blow | 1795.084 | 14 |  | 0 | 0 |  | 0 | 0 |
| floul | soul | 39.16201 | 1 | foul | 9.72067 | 1 | ghoul | 0.670391 | 1 |  | 0 | 0 |
| stook | book | 1380.335 | 10 | spook | 0.726257 | 2 |  | 0 | 0 |  | 0 | 0 |
| plood | brood | 295.8659 | 5 | good | 1153.24 | 4 | blood | 154.7486 | 2 |  | 0 | 0 |
| prear | dear | 1383.464 | 14 | bear | 165.8101 | 5 |  | 0 | 0 |  | 0 | 0 |
| phreat | beat | 553.9106 | 15 | sweat | 89.16201 | 2 | great | 612.9609 | 1 |  | 0 | 0 |
| spow | brow | 2754.358 | 14 | blow | 1795.084 | 14 |  | 0 | 0 |  | 0 | 0 |
| gow | brow | 2754.358 | 14 | blow | 1795.084 | 14 |  | 0 | 0 |  | 0 | 0 |
| brear | dear | 1383.464 | 14 | bear | 165.8101 | 5 |  | 0 | 0 |  | 0 | 0 |
| fush | gush | 81.73184 | 13 | bush | 84.13408 | 3 |  | 0 | 0 |  | 0 | 0 |
| meast | beast | 500.2793 | 5 | breast | 43.12849 | 1 |  | 0 | 0 |  | 0 | 0 |
| poad | load | 223.9665 | 5 | broad | 40.50279 | 1 |  | 0 | 0 |  | 0 | 0 |
| vey | hey | 4551.732 | 8 | key | 69.44134 | 1 |  | 0 | 0 |  | 0 | 0 |
| hase | base | 431.5084 | 3 | phase | 60.44693 | 2 | vase | 3.96648 | 1 |  | 0 | 0 |
| steese | cheese | 27.3743 | 1 | geese | 4.748603 | 1 |  | 0 | 0 |  | 0 | 0 |
| sphass | crass | 123.4078 | 6 | brass | 507.095 | 5 |  | 0 | 0 |  | 0 | 0 |
| cown | blown | 1314.581 | 9 | brown | 1492.011 | 8 |  | 0 | 0 |  | 0 | 0 |
| rost | host | 1229.609 | 4 | cost | 345.3073 | 3 | dost | 0.614525 | 1 |  | 0 | 0 |
| frood | brood | 295.8659 | 5 | good | 1153.24 | 4 | blood | 154.7486 | 2 |  | 0 | 0 |
| shost | host | 1229.609 | 4 | cost | 345.3073 | 3 |  | 0 | 0 |  | 0 | 0 |
| poul | soul | 39.16201 | 1 | foul | 9.72067 | 1 | ghoul | 0.670391 | 1 |  | 0 | 0 |
| las | has | 8473.184 | 2 | bras | 1.284916 | 2 | gas | 65.02793 | 1 | was | 10376.54 | 1 |
| brove | cove | 96.48045 | 13 | love | 353.4637 | 4 | move | 232.1229 | 2 |  | 0 | 0 |
| deight | weight | 187.5419 | 3 | height | 32.90503 | 1 |  | 0 | 0 |  | 0 | 0 |
| pimb | climb | 35.13966 | 1 | limb | 7.430168 | 1 |  | 0 | 0 |  | 0 | 0 |
| pove | cove | 96.48045 | 13 | love | 353.4637 | 4 | move | 232.1229 | 2 |  | 0 | 0 |
| meese | cheese | 27.3743 | 1 | geese | 4.748603 | 1 |  | 0 | 0 |  | 0 | 0 |
| sonk | honk | 0.782123 | 3 | monk | 3.743017 | 1 |  | 0 | 0 |  | 0 | 0 |
| bouth | mouth | 320.4469 | 2 | youth | 59.72067 | 2 |  | 0 | 0 |  | 0 | 0 |
| poot | boot | 59.55307 | 9 | foot | 112.2905 | 2 |  | 0 | 0 |  | 0 | 0 |
| briend | friend | 164.4693 | 1 | fiend | 1.22905 | 1 |  | 0 | 0 |  | 0 | 0 |
| sint | mint | 68.65922 | 12 | pint | 9.441341 | 1 |  | 0 | 0 |  | 0 | 0 |
| kear | dear | 1383.464 | 14 | bear | 165.8101 | 5 |  | 0 | 0 |  | 0 | 0 |
| fass | crass | 123.4078 | 6 | brass | 507.095 | 5 |  | 0 | 0 |  | 0 | 0 |
| froll | roll | 30.44693 | 4 | doll | 26.48045 | 4 |  | 0 | 0 |  | 0 | 0 |
| gath | bath | 117.2067 | 7 | math | 5.921788 | 3 | wrath | 7.039106 | 1 |  | 0 | 0 |
| wough | rough | 80.22346 | 4 | bough | 11.22905 | 4 | dough | 908.1006 | 2 | cough | 11.06145 | 1 |
| lon | con | 6539.441 | 5 | son | 227.933 | 3 |  | 0 | 0 |  | 0 | 0 |
| sni | chi | 12.01117 | 4 | ski | 10 | 4 |  | 0 | 0 |  | 0 | 0 |
| choe | doe | 33.63128 | 8 | shoe | 14.13408 | 1 |  | 0 | 0 |  | 0 | 0 |
| clow | brow | 2754.358 | 14 | blow | 1795.084 | 14 |  | 0 | 0 |  | 0 | 0 |
| goot | boot | 59.55307 | 9 | foot | 112.2905 | 2 |  | 0 | 0 |  | 0 | 0 |
| swere | here | 761.0056 | 3 | there | 4006.536 | 2 | were | 3370.726 | 1 |  | 0 | 0 |
| fleak | beak | 221.3966 | 13 | break | 105.2514 | 2 |  | 0 | 0 |  | 0 | 0 |
| gleat | beat | 553.9106 | 15 | sweat | 89.16201 | 2 | great | 612.9609 | 1 |  | 0 | 0 |
| skow | brow | 2754.358 | 14 | blow | 1795.084 | 14 |  | 0 | 0 |  | 0 | 0 |
| pown | blown | 1314.581 | 9 | brown | 1492.011 | 8 |  | 0 | 0 |  | 0 | 0 |
| tase | base | 431.5084 | 3 | phase | 60.44693 | 2 | vase | 3.96648 | 1 |  | 0 | 0 |
| throod | brood | 295.8659 | 5 | good | 1153.24 | 4 | blood | 154.7486 | 2 |  | 0 | 0 |
